# Recombinant Tissue Plasminogen Activator Induces Neurological Side Effects Independent on Thrombolysis in Mechanical Animal Models of Focal Cerebral Infarction: A Systematic Review and Meta-Analysis

**DOI:** 10.1371/journal.pone.0158848

**Published:** 2016-07-07

**Authors:** Mei-Xue Dong, Qing-Chuan Hu, Peng Shen, Jun-Xi Pan, You-Dong Wei, Yi-Yun Liu, Yi-Fei Ren, Zi-Hong Liang, Hai-Yang Wang, Li-Bo Zhao, Peng Xie

**Affiliations:** 1 Department of Neurology, the First Affiliated Hospital of Chongqing Medical University, Chongqing, China; 2 Institute of Neuroscience and the Collaborative Innovation Center for Brain Science, Chongqing Medical University, Chongqing, China; 3 Chongqing Key Laboratory of Neurobiology, Chongqing, China; 4 Department of Neurology, Yongchuan Hospital of Chongqing Medical University, Chongqing, China; Massachusetts General Hospital/Harvard Medical School, UNITED STATES

## Abstract

**Background and Purpose:**

Recombinant tissue plasminogen activator (rtPA) is the only effective drug approved by US FDA to treat ischemic stroke, and it contains pleiotropic effects besides thrombolysis. We performed a meta-analysis to clarify effect of tissue plasminogen activator (tPA) on cerebral infarction besides its thrombolysis property in mechanical animal stroke.

**Methods:**

Relevant studies were identified by two reviewers after searching online databases, including Pubmed, Embase, and ScienceDirect, from 1979 to 2016. We identified 6, 65, 17, 12, 16, 12 and 13 comparisons reporting effect of endogenous tPA on infarction volume and effects of rtPA on infarction volume, blood-brain barrier, brain edema, intracerebral hemorrhage, neurological function and mortality rate in all 47 included studies. Standardized mean differences for continuous measures and risk ratio for dichotomous measures were calculated to assess the effects of endogenous tPA and rtPA on cerebral infarction in animals. The quality of included studies was assessed using the Stroke Therapy Academic Industry Roundtable score. Subgroup analysis, meta-regression and sensitivity analysis were performed to explore sources of heterogeneity. Funnel plot, Trim and Fill method and Egger’s test were obtained to detect publication bias.

**Results:**

We found that both endogenous tPA and rtPA had not enlarged infarction volume, or deteriorated neurological function. However, rtPA would disrupt blood-brain barrier, aggravate brain edema, induce intracerebral hemorrhage and increase mortality rate.

**Conclusions:**

This meta-analysis reveals rtPA can lead to neurological side effects besides thrombolysis in mechanical animal stroke, which may account for clinical exacerbation for stroke patients that do not achieve vascular recanalization with rtPA.

## Introduction

Acute cerebral infarction is a major cause of adult mortality and disability, and its incidence will grow as population age[1, 2]. It still remains as a serious and significant global health problem in industrialized countries[3]. Thrombolysis or clot-dissolving treatment is the most effective treatment till now and significantly reduces the risk of long-term dependency on others for daily activities in spite of an increased risk of bleeding in the brain[4]. Recombinant tissue plasminogen activator (rtPA), a serine protease that converts the proenzyme plasminogen into the proteinase plasmin, is the only effective thrombolytic drug for patients with acute cerebral infarction approved by US FDA since 1996[5, 6]. RtPA is undoubtedly an effective drug in clinic[7, 8] while its common well-known side effects are bleeding[9], reperfusion injury with edema, and angioedema after clot dissolving[9, 10]. In addition to its thrombolytic property, rtPA can act upon the brain parenchyma leading to seizures and neurotoxicity according to some researches[11]. Lots of researchers revealed that rtPA can induce excitotoxic neuronal degeneration in vitro [12], degrade blood-brain barrier (BBB)[13, 14], and potentiate ischemic brain injury in stroke model[15]. They argued that rtPA had caused unexpected side effects independent on its thrombolysis property in mechanical animal stroke and neurological function of patients who do not achieve vascular recanalization with rtPA had significantly deteriorated. They found that knockout mice deficient in tissue plasminogen activator (tPA) were protected against cerebral ischemia[16, 17], and tPA variant provided a novel approach for limiting neuronal toxicity caused by the increased endogenous tPA and glutamate following traumatic brain injury[18]. Meanwhile, some researchers hold the viewpoint that rtPA is not only a thrombolytic but also neuroprotective drug, whilst rtPA can protect neurons independent on its thrombolytic property[19, 20]. The evidence was that tPA can also slow down Alzheimer’s Disease-related pathology development in APPswe/PS1 mice[21, 22], induce early hypoxic or ischemic tolerance by increasing the expression of neuronal tumor necrosis factor-α[23] and protect neuronal survival through inducing the uptake of glucose in the ischemic brain[24]. As we know, neuroprotection is an important supplementary treatment, and it remains as an urgent need to develop neuroprotective drugs improving the quality of life for patients with cerebral infarction[25]. We need to confirm whether rtPA is a drug containing neurotoxic or neuroprotective function independent on thrombolysis in ischemic stroke for further treatment strategy. Thus, a pre-clinical meta-analysis of animal studies was performed to clarify side effect of tPA on cerebral infarction and studies using mechanical stroke model were retrieved here to avoid thrombolysis property of tPA.

## Materials and Methods

### Data sources and searches

Pubmed, Embase and ScienceDirect were searched from January 1, 1979 to January 1, 2016 without language restrictions as follows: (“thrombolysis” OR “thrombolytic” OR “tpa” OR “t-pa” OR “rtpa” OR “rt-pa” OR “tissue plasminogen activator” OR “alteplase” OR “activacin” OR “actilyse” OR “activase” OR “grtpa”) AND (“stroke” OR “ischemia” OR “ischemic” OR “cerebrovascular” OR “middle cerebral artery” OR “MCA” OR “ACA” OR “anterior cerebral artery” OR “MCAO”) AND (“rat*” OR “mouse” OR “mice” OR “rabbit*” OR “rodent*” OR “animal*”). Other potential studies were identified by consulting previous reviews and reference lists of retrieved records.

### Inclusion and exclusion criteria

The inclusion criteria were as follows: (i) using a filament or ligation mechanical stroke model; (ii) containing both rtPA and saline groups in non-transgenic animals, or both tPA deficient and wild-type groups in transgenic animals; (iii) reported means and their standard errors (S.E.) or standard deviations (S.D.) of infarction volume in the text. The exclusion criteria were as follows: (i) photochemical thrombosis or thromboembolic stroke model; (ii) review; (iii) conference abstract; (iv) human study; (v) dose of rtPA administration > 10mg/kg or timing of rtPA >6 hours.

### Data extraction and quality assessment

Data were extracted independently by two investigators (M.X.D. and Q.C.H.), and any differences were resolved by discussion with a third investigator (P.S.). We retrieved the following parameters from each included study: first author’s name, publication year, species, ischemic model, duration of ischemia, dose of rtPA administration, timing of rtPA, timing of assessment, infarction volume, blood-brain barrier, brain edema, intracerebral hemorrhage, neurological deficit sore, mortality rate, evaluation methodology and number of animals. Means, S.E. and S.D. for continuous measures were extracted from the text where possible or by use of a screen grab tool when they were represented in diagrammatic form[26]. S.E. can be changed to S.D. using the following formulas: S.D. = S.E.* sqrt(n). Dichotomous data were extracted from the text in table. We used the Stroke Therapy Academic Industry Roundtable score (STAIR) to assess the study quality in this meta-analysis[27].

### Statistical methods

Infarction volume was the primary efficacy outcome while BBB, brain edema, intracerebral hemorrhage, neurological deficit sore and mortality rate were the secondary efficacy outcomes. Statistical analysis process was described as before[28–31]. Briefly, standardized mean differences (SMDs) were calculated to assess changes of each efficacy outcome for continuous measures and combined into a pooled summary SMD using a random-effect model. Risk ratios (RRs) were calculated for dichotomous measures using Mantel-Haenszel statistical method and random-effect model. Heterogeneity across studies was assessed using Chi^2^ test and *I*^2^ statistic. An *I*^2^ of <25%, <50%, <75% and > = 75% represented low, moderate, high and extremely high heterogeneity, respectively. A meta-regression model was used to detect potential heterogeneity between the included studies based on moderators such as species, model, duration of ischemia, dose of rtPA administration, timing of assessment, timing of rtPA and STAIR score. Subgroup analyses of primary efficacy outcome were performed based on species (mouse versus rat), model (filament versus MCAO ligation), duration of ischemia (permanent versus transient), timing of rtPA (< = 3 hours versus 3~4.5 hours versus 4.5~6 hours), dose of rtPA administration (<10mg/kg versus 10mg/kg), evaluation methodology, and STAIR score (< = 3 versus > = 4). A sensitivity analysis was conducted using the leave-one-out method. Furthermore, publication bias was assessed using funnel plot, Trim and Fill method and Egger’s test. Data were analyzed using the RevMan5.3 (Cochrane Information Management System), Stata version12.0 (Stata Corp, College Station, Texas, USA) and R version3.2.4. (www.r-project.org).

## Results

### Literature search results

**Fig 1:**
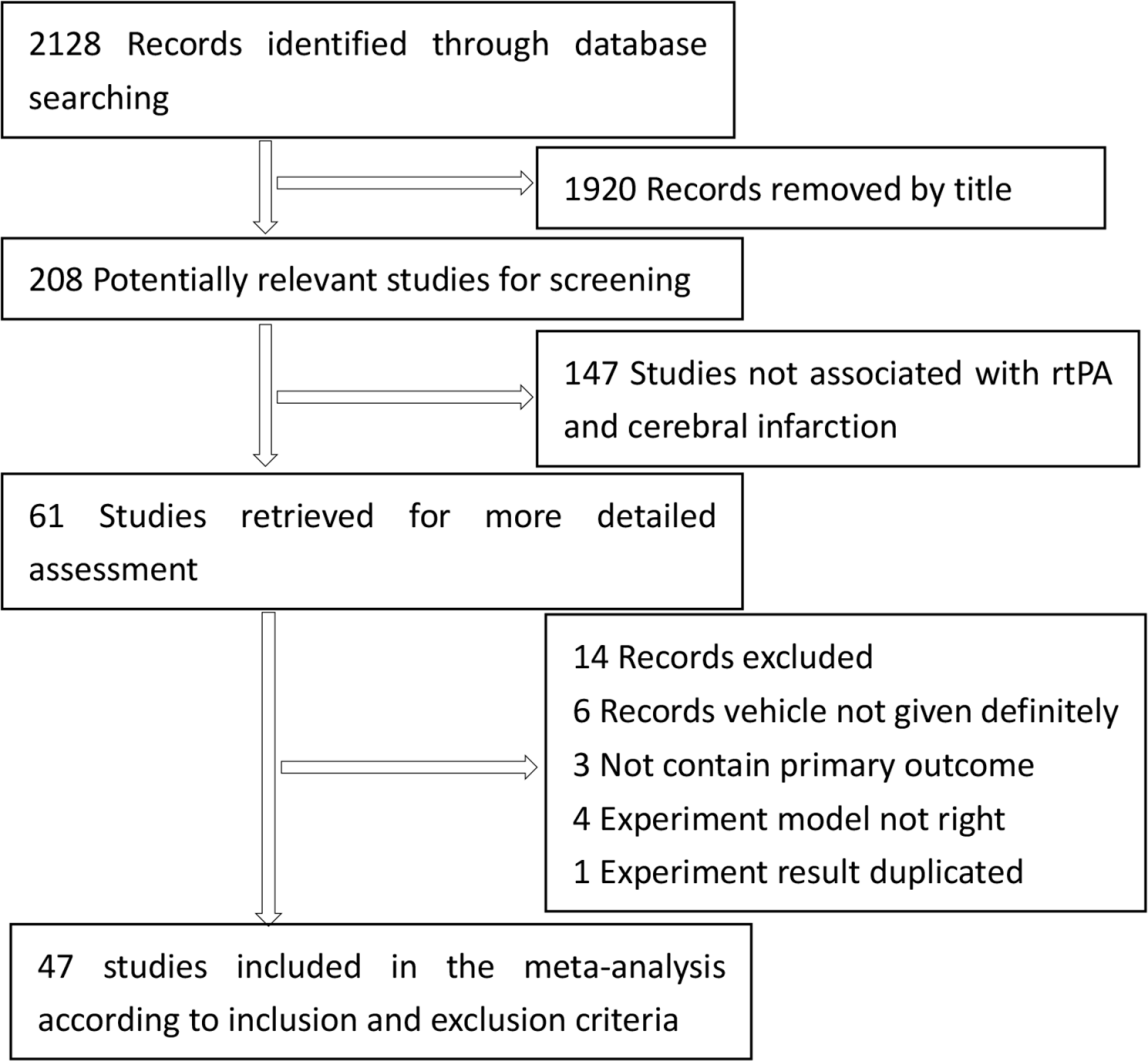
Flow chart of study selection.

The detailed flowchart of study selection was shown in Fig 1. A total of 2128 records were initially identified; of these, 1920 records were excluded by title/abstract screening. Of the 208 potentially relevant records, 147 records were excluded because of stroke models, research purposes, reviews or repeated reports. Fourteen additional records were further excluded for the following reasons: vehicle of control group was not given definitely in 6 records, primary efficacy outcome was not given definitely in 3 records, stroke model of 4 records did not accord with the inclusion criteria, and 1 record had duplicated. Thus, 47 studies[13, 32–77] were finally included in this meta-analysis.

### Study characteristics

A summary of the characteristics of the included studies was shown in Table 1. The whole 47 studies were published from 1998 to 2015, the experiment objects of 25 studies were rats and 23 studies were mice, and only 5 studies used tPA deficient mice. Forty one studies adopted filament model and 6 studies adopted MCAO ligation model. Duration of ischemia was from 60 minutes to permanent and timing of rtPA was from 15 minutes to 360 minutes. Most of the studies adopted rtPA at a dose of 10 mg/kg and the others adopted lower doses. Infarction volume was calculated by methods of 2,3,5-triphenyltetrazolium chloride, hematoxylin eosin staining, cresyl violet or MAP-2 antibody staining. BBB was assessed by methods of Evans blue or IgG extravasation. Brain edema was obtained using the following formulas: i) (volume of ipsilateral hemisphere–volume of contralateral hemisphere) /volume of contralateral hemisphere; or ii) (wet weight–dry weight) /wet weight. Intracerebral hemorrhage was acquired from figuring up hemorrhagic volume, hemorrhagic score, or detecting hemoglobin content by spectrophotometric assay or western blotting. Neurological function was exhibited as neurological deficit scoring or Benderson test. Study quality was from 2 to 6 stars assessed using STAIR score (Table 2).

**Table 1:** Summary and characteristics of included studies.

*No*.	*First author*	*Publication year*	*Species*	*Ischemic model*	*DI (min)*	*Time (min)*	*TA(h)*	*Dose (mg/kg)*
*1*	*Wang(32)*	*1998*	*Wild-type and tPA deficient male mice on C57BL/6 and SV129 backgrounds*, *23-27g*	*Filament*	*120*,*180*	*-*	*24*	*-*
*2*	*Killic(33)*	*1999*	*Adult C57BL/6 mice*, *21-27g*	*Filament*	*90*	*15*	*24*	*10*
*3*	*Klein(34)*	*1999*	*Male spontaneously hypertensive Wistar rats*, *200g*	*MCAO ligation*	*120*	*120*	*24*	*10*
*4*	*Meng(35)*	*1999*	*SD rats*, *270-310g*	*Filament*	*120*	*120*	*24*	*10*
*5*	*Tabrizi(36)*	*1999*	*Wild-type and tPA deficient male mice on mixed 129/Sv and C57BL/6 backgrounds*, *22-27g*	*Filament*	*180*	*-*	*24*	*-*
*6*	*Killic(37)*	*2001*	*Adult male C57BL/6 mice*, *21-28g*	*Filament*	*90*	*90*	*24*	*0*.*2*,*1*,*2*,*10*
*7*	*Gautier(38)*	*2003*	*Male spontaneously hypertensive or Wistar rats*, *270-320g*	*Filament*	*60*	*360*	*24*	*3*,*10*
*8*	*Zhang(39)*	*2004*	*Adult male Wistar rats*, *250-280g*	*Filament*	*90*	*90*	*168*	*5*
*9*	*Grobholz(40)*	*2005*	*Male Wistar rats*, *250-350g*	*Filament*	*180*	*180*	*24*	*0*.*9*,*9*
*10*	*Tsuji(43)*	*2005*	*Male spontaneously hypertensive or Wistar rats*, *260-280g*	*Filament*	*180*	*180*	*24*	*10*
*11*	*Killic(41)*	*2005a*	*Male C57BL/6 mice*, *21-26g*	*Filament*	*90*	*90*	*24*	*10*
*12*	*Killic(42)*	*2005b*	*Male C57BL/6 mice*, *21-26g*	*Filament*	*90*	*90*	*24*	*10*
*13*	*Armstead(44)*	*2006*	*SD rats*, *250g*	*Filament*	*120*	*120*	*24*	*6*
*14*	*Burggraf(45)*	*2007*	*Male Wistar rats*, *250-350g*	*Filament*	*180*	*150*	*24*	*0*.*9*,*9*
*15*	*Armugam(46)*	*2009*	*Male SD rats*, *200-300g*	*Filament*	*60*	*30*	*24*	*10*
*16*	*Lu(47)*	*2009*	*Male SD rats*, *290-340g*	*Filament*	*300*	*300*	*24*	*1*
*17*	*Machado(48)*	*2009*	*Male Wistar rats*, *270-300g*	*Filament*	*180*	*180*	*24*	*10*
*18*	*Oka(49)*	*2009*	*Adult male SD rats*, *250-300g*	*Filament*	*60*	*60*	*24*	*10*
*19*	*Roussel(50)*	*2009*	*Wild-type and tPA deficient mice on C57BL/6 background*, *4 months*	*MCAO ligation*	*Permanent*	*-*	*48*	*-*
*20*	*Tang(51)*	*2009*	*Male SD rats*, *280-350g*	*Filament*	*120*	*120*	*24*	*10*
*21*	*Yagi(52)*	*2009*	*Male Wistar rats*, *250-280g*	*Filament*	*180*	*180*	*24*	*10*
*22*	*Abu(53)*	*2010*	*SD rats*	*Filament*	*60*,*180*	*120*	*24*	*6*
*23*	*Burggraf(54)*	*2010*	*Male Wistar rats*, *250-300g*	*Filament*	*180*	*150*	*24*	*9*
*24*	*Ishiguro(55)*	*2010*	*Male ddY mice*, *4 weeks*, *22-28g*	*Filament*	*120*,*180*,*360*	*120*,*180*,*360*	*24*	*10*
*25*	*Wu(56)*	*2010*	*Male wild-type and tPA deficient mice on C57BL/6 background*, *8–12 weeks*	*Filament*	*30*	*-*	*24*	*-*
*26*	*Zechariah(57)*	*2010*	*Adult male C57BL/6 mice*, *20-25g*	*Filament*	*90*	*90*	*24*	*10*
*27*	*Berny(58)*	*2011*	*C57BL/6 mice*, *21-27g*, *3 months*	*Filament*	*60*	*15*	*24*	*2*.*5*
*28*	*Crumrine(59)*	*2011*	*Adult male spontaneously hypertensive rats*, *330-380g*	*MCAO ligation*	*360*	*300*,*360*	*24*	*1*,*5*,*10*
*29*	*Shen(60)*	*2011*	*Adult male wild-type and tPA deficient mice on C57BL/6 background*, *22-25g*	*Filament*	*Permanent*	*-*	*336*	*-*
*30*	*Crumrine(61)*	*2012*	*Adult male spontaneously hypertensive rats*, *330-380g*	*MCAO ligation*	*360*	*300*	*24*	*10*
*31*	*Deguchi(62)*	*2012*	*Adult male Wistar rats*, *250-280g*, *12 weeks*	*Filament*	*90*	*90*	*96*	*10*
*32*	*Ishiguro(63)*	*2012*	*Male ddY mice*, *4 weeks*, *22-28g*	*Filament*	*360*	*360*	*24*	*10*
*33*	*Turner(64)*	*2012*	*Adult male SD rats*, *365-395g*	*Filament*	*120*	*120*	*24*	*1*
*34*	*Wu(65)*	*2012*	*Male C57BL/6 mice*	*Filament*	*60*	*120*	*24*	*1*,*4*.*5*,*9*
*35*	*Crawley(66)*	*2013*	*Adult male C57BL/6 mice*	*Filament*	*60*	*180*	*24*	*10*
*36*	*Haddad(67)*	*2013*	*Male Swiss mice*, *27-32g*	*Filament*	*Permanent*	*360*	*48*	*10*
*37*	*Sutherland(68)*	*2013*	*Male Wistar rats*, *243-338g*	*Filament*	*90*	*90*	*24*	*10*
*38*	*Tang(69)*	*2013*	*Male C57BL/6 mice*, *25-30g*	*Filament*	*60*	*60*	*24*	*10*
*39*	*Teng(13)*	*2013*	*Male Swiss mice*, *27-32g*	*Filament*	*Permanent*	*360*	*24*	*10*
*40*	*Lenglet(70)*	*2014*	*Male 129/SvEV mice*, *3–4 months*, *25*.*1g*	*Filament*	*60*	*60*	*6*,*24*,*72*	*10*
*41*	*Won(71)*	*2014*	*Male SD rats*, *3 months*, *300-350g*	*Filament*	*270*	*260*	*24*	*5*
*42*	*Zhu(72)*	*2014*	*Male DR2-Tg mice*, *8–12 weeks*, *20*.*1–27*.*7g*	*Filament*	*60*	*15*	*24*,*72*	*10*
*43*	*Allahtavakoli(73)*	*2015*	*Male rats*, *200-250g*	*MCAO ligation*	*Permanent*	*300*	*48*	*1*
*44*	*Cechmanek(74)*	*2015*	*Male C57BL/6 mice*, *25-30g*, *3 months*	*Filament*	*30*	*30*	*72*	*10*
*45*	*Kocic(75)*	*2015*	*Male Wistar rats*, *270-350g*	*Filament*	*Permanent*	*120*	*168*	*10*
*46*	*Liang(76)*	*2015*	*Male SD rats*, *290-320g*	*MCAO ligation*	*180*	*180*,*300*,*420*	*5*,*7*,*9*	*10*
*47*	*Nakano(77)*	*2015*	*Male ddY mice*, *6–8 weeks*, *25-35g*	*Filament*	*120*	*120*	*24*	*10*

No., number; DI, duration of ischemia; min, minute; h, hour; Time, timing of rtPA; TA, timing of assessment; Dose, dose of rtPA administration; MCAO, middle cerebral artery occlusion; SD, Sprague Dawley; tPA, tissue plasminogen activator.

**Table 2:** Stroke Therapy Academic Industry Roundtable (STAIR) score of included studies.

*No*.	*First author*	*Year*	*Size*	*Crit*	*Rand*	*conce*	*Exclu*	*Blind*	*Confli*	*Total*
*1*	*Wang (32)*	*1998*	*0*	*1*	*0*	*0*	*1*	*0*	*1*	*3*
*2*	*Killic (33)*	*1999*	*0*	*1*	*0*	*0*	*1*	*0*	*0*	*2*
*3*	*Klein (34)*	*1999*	*1*	*1*	*1*	*0*	*1*	*0*	*0*	*4*
*4*	*Meng (35)*	*1999*	*0*	*1*	*0*	*0*	*1*	*0*	*1*	*3*
*5*	*Tabrizi (36)*	*1999*	*0*	*1*	*0*	*0*	*1*	*1*	*1*	*4*
*6*	*Killic (37)*	*2001*	*0*	*1*	*0*	*0*	*1*	*0*	*1*	*3*
*7*	*Gautier (38)*	*2003*	*0*	*1*	*0*	*0*	*1*	*1*	*1*	*4*
*8*	*Zhang (39)*	*2004*	*0*	*1*	*1*	*0*	*1*	*0*	*1*	*4*
*9*	*Grobholz (40)*	*2005*	*0*	*1*	*0*	*0*	*1*	*0*	*1*	*3*
*10*	*Tsuji (41)*	*2005*	*0*	*1*	*0*	*0*	*1*	*0*	*1*	*3*
*11*	*Killic (42)*	*2005a*	*0*	*1*	*0*	*0*	*1*	*0*	*1*	*3*
*12*	*Killic (43)*	*2005b*	*0*	*1*	*0*	*0*	*1*	*0*	*1*	*3*
*13*	*Armstead (44)*	*2006*	*0*	*1*	*0*	*0*	*1*	*0*	*1*	*3*
*14*	*Burggraf (45)*	*2007*	*0*	*1*	*0*	*0*	*1*	*0*	*1*	*3*
*15*	*Armugam (46)*	*2009*	*0*	*1*	*0*	*0*	*1*	*0*	*1*	*3*
*16*	*Lu (47)*	*2009*	*0*	*1*	*1*	*1*	*1*	*1*	*1*	*6*
*17*	*Machado (48)*	*2009*	*0*	*1*	*0*	*0*	*1*	*0*	*1*	*3*
*18*	*Oka (49)*	*2009*	*0*	*1*	*1*	*0*	*1*	*1*	*1*	*5*
*19*	*Roussel (50)*	*2009*	*0*	*1*	*1*	*0*	*1*	*0*	*1*	*4*
*20*	*Tang (51)*	*2009*	*0*	*1*	*1*	*1*	*1*	*1*	*0*	*5*
*21*	*Yagi (52)*	*2009*	*0*	*1*	*0*	*0*	*1*	*0*	*1*	*3*
*22*	*Abu (53)*	*2010*	*0*	*1*	*1*	*1*	*1*	*1*	*1*	*6*
*23*	*Burggraf (54)*	*2010*	*0*	*1*	*0*	*0*	*1*	*0*	*1*	*3*
*24*	*Ishiguro (55)*	*2010*	*0*	*1*	*1*	*0*	*1*	*1*	*0*	*4*
*25*	*Wu (56)*	*2010*	*0*	*1*	*0*	*0*	*1*	*0*	*1*	*3*
*26*	*Zechariah (57)*	*2010*	*0*	*1*	*0*	*0*	*1*	*0*	*1*	*3*
*27*	*Berny (58)*	*2011*	*0*	*1*	*1*	*0*	*1*	*0*	*1*	*4*
*28*	*Crumrine (59)*	*2011*	*0*	*1*	*1*	*0*	*1*	*1*	*1*	*5*
*29*	*Shen (60)*	*2011*	*0*	*1*	*1*	*0*	*1*	*1*	*1*	*5*
*30*	*Crumrine (61)*	*2012*	*0*	*1*	*1*	*0*	*1*	*1*	*1*	*5*
*31*	*Deguchi (62)*	*2012*	*0*	*1*	*0*	*0*	*1*	*0*	*1*	*3*
*32*	*Ishiguro (63)*	*2012*	*0*	*1*	*1*	*0*	*1*	*0*	*0*	*3*
*33*	*Turner (64)*	*2012*	*0*	*1*	*1*	*1*	*1*	*1*	*1*	*6*
*34*	*Wu (65)*	*2012*	*0*	*1*	*0*	*0*	*1*	*0*	*1*	*3*
*35*	*Crawley (66)*	*2013*	*0*	*1*	*1*	*0*	*1*	*1*	*1*	*5*
*36*	*Haddad (67)*	*2013*	*0*	*1*	*1*	*0*	*1*	*1*	*1*	*5*
*37*	*Sutherland (68)*	*2013*	*0*	*1*	*1*	*1*	*1*	*1*	*1*	*6*
*38*	*Tang (69)*	*2013*	*0*	*1*	*1*	*1*	*1*	*1*	*1*	*6*
*39*	*Teng (13)*	*2013*	*0*	*1*	*1*	*0*	*1*	*0*	*1*	*4*
*40*	*Lenglet (70*	*2014*	*0*	*1*	*1*	*0*	*1*	*1*	*0*	*4*
*41*	*Won (71)*	*2014*	*0*	*1*	*1*	*1*	*1*	*1*	*1*	*6*
*42*	*Zhu (72)*	*2014*	*0*	*1*	*1*	*0*	*1*	*0*	*1*	*4*
*43*	*Allahtavakoli (73)*	*2015*	*0*	*1*	*1*	*1*	*1*	*0*	*1*	*5*
*44*	*Cechmanek (74)*	*2015*	*0*	*1*	*1*	*0*	*1*	*0*	*1*	*4*
*45*	*Kocic (75)*	*2015*	*0*	*1*	*0*	*0*	*1*	*0*	*1*	*3*
*46*	*Liang (76)*	*2015*	*0*	*1*	*1*	*0*	*1*	*1*	*1*	*5*
*47*	*Nakano (77)*	*2015*	*0*	*1*	*0*	*0*	*1*	*0*	*0*	*2*

No., number; Size, sample size calculation; Crit, inclusion and exclusion criteria; rand, randomization; conce, allocation concealment; exclu, reporting of animals excluded from analysis; blind, blinded assessment of outcome; confli, reporting potential conflicts of interest and study funding; total, total score of STAIR.

### Effect of rtPA on infarction volume

**Fig 2:**
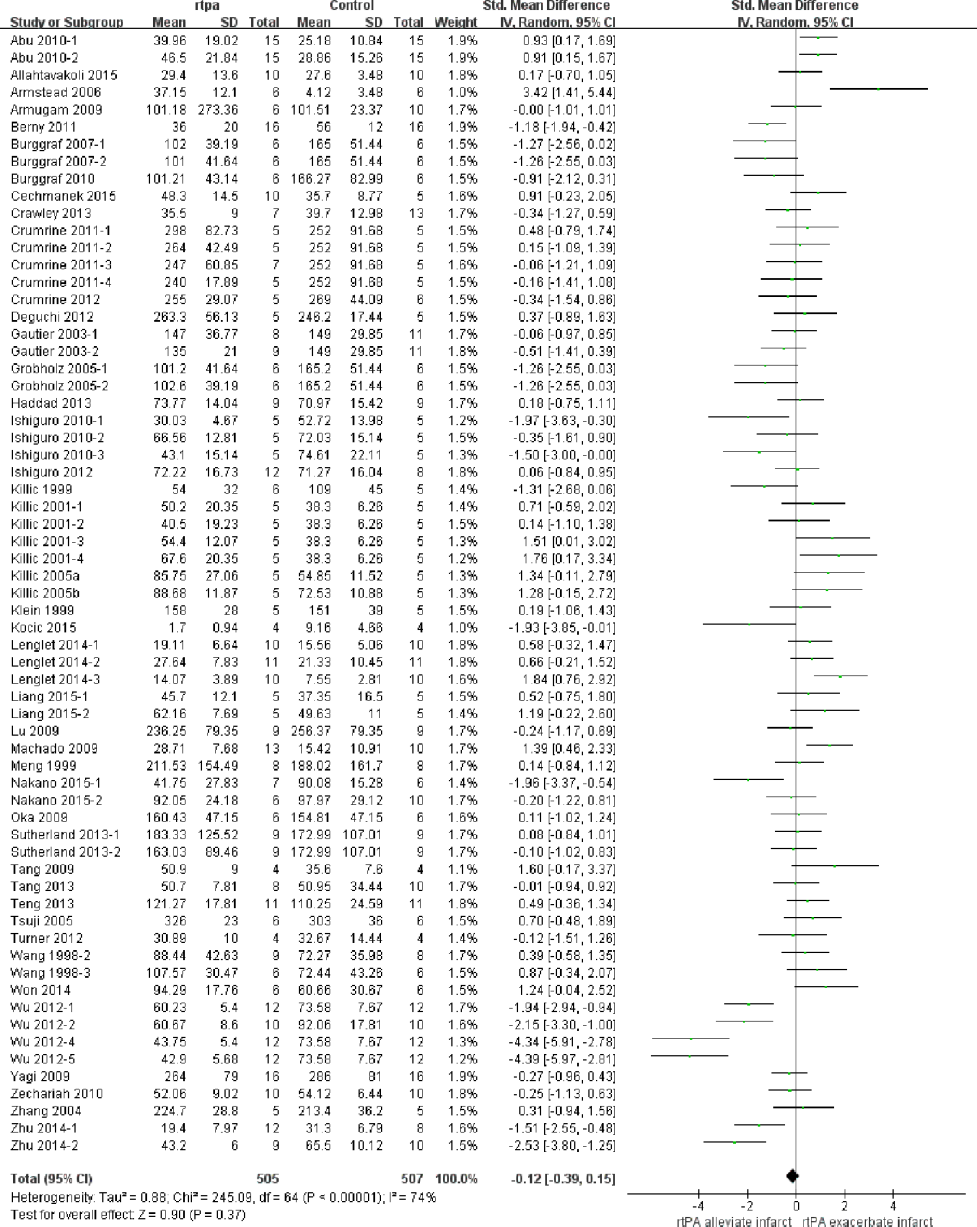
Forest plot of SMDs of rtPA’s effect on infarction volume. Data of all studies and the pooled effect across all studies were provided. The overall effect was not significant (*p* = 0.37) and heterogeneity was high (*I*^*2*^ = 74%). SMD, standardized mean difference.

**Fig 3:**
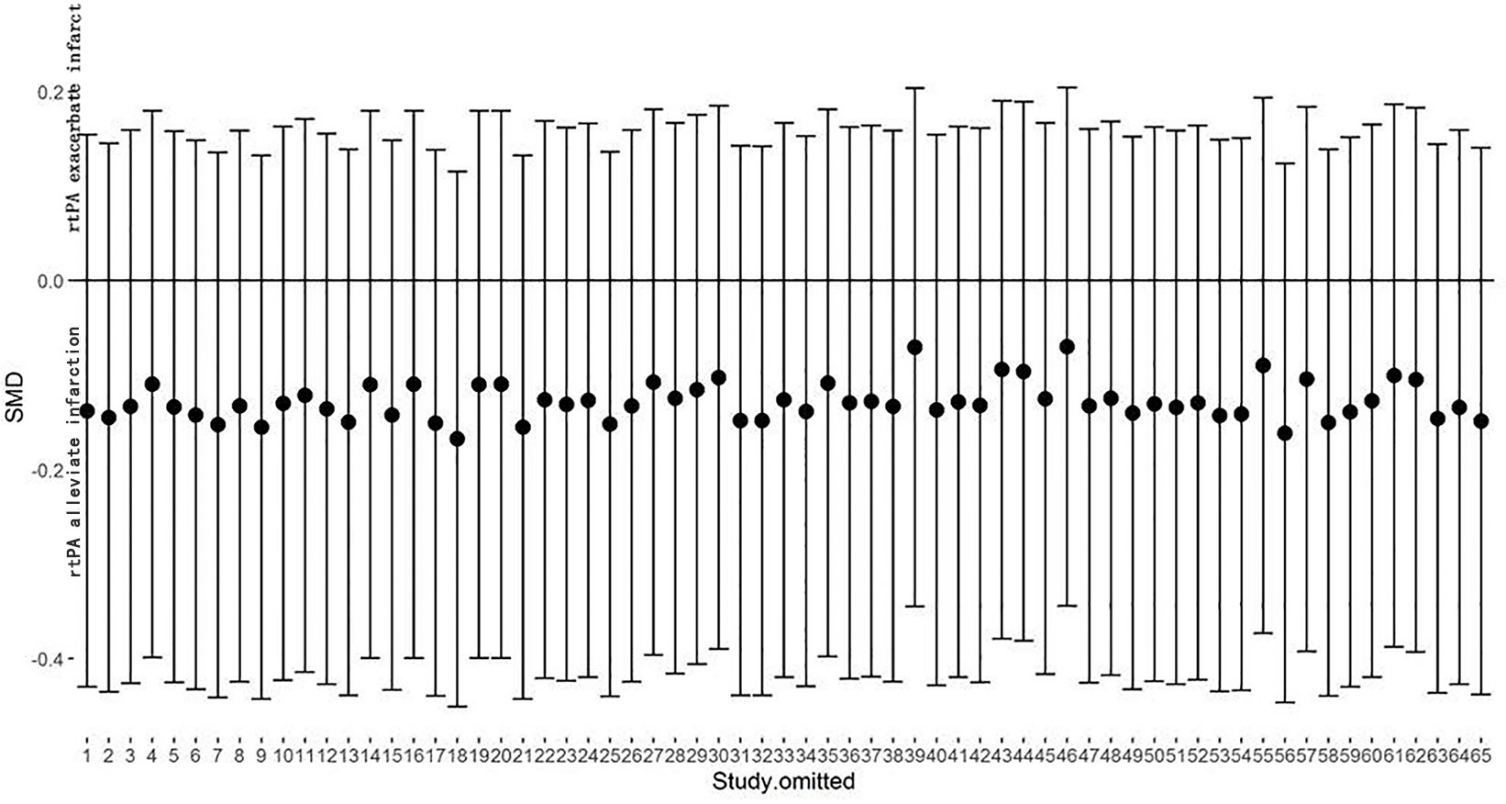
Sensitivity meta-analyses of rtPA’s effect on infarction volume. The figure showed all 95%CI of SMDs after omitting each study as vertical line. The results remained stable using the leave-one-out method. CI, confidence interval; SMD, standardized mean difference.

**Fig 4:**
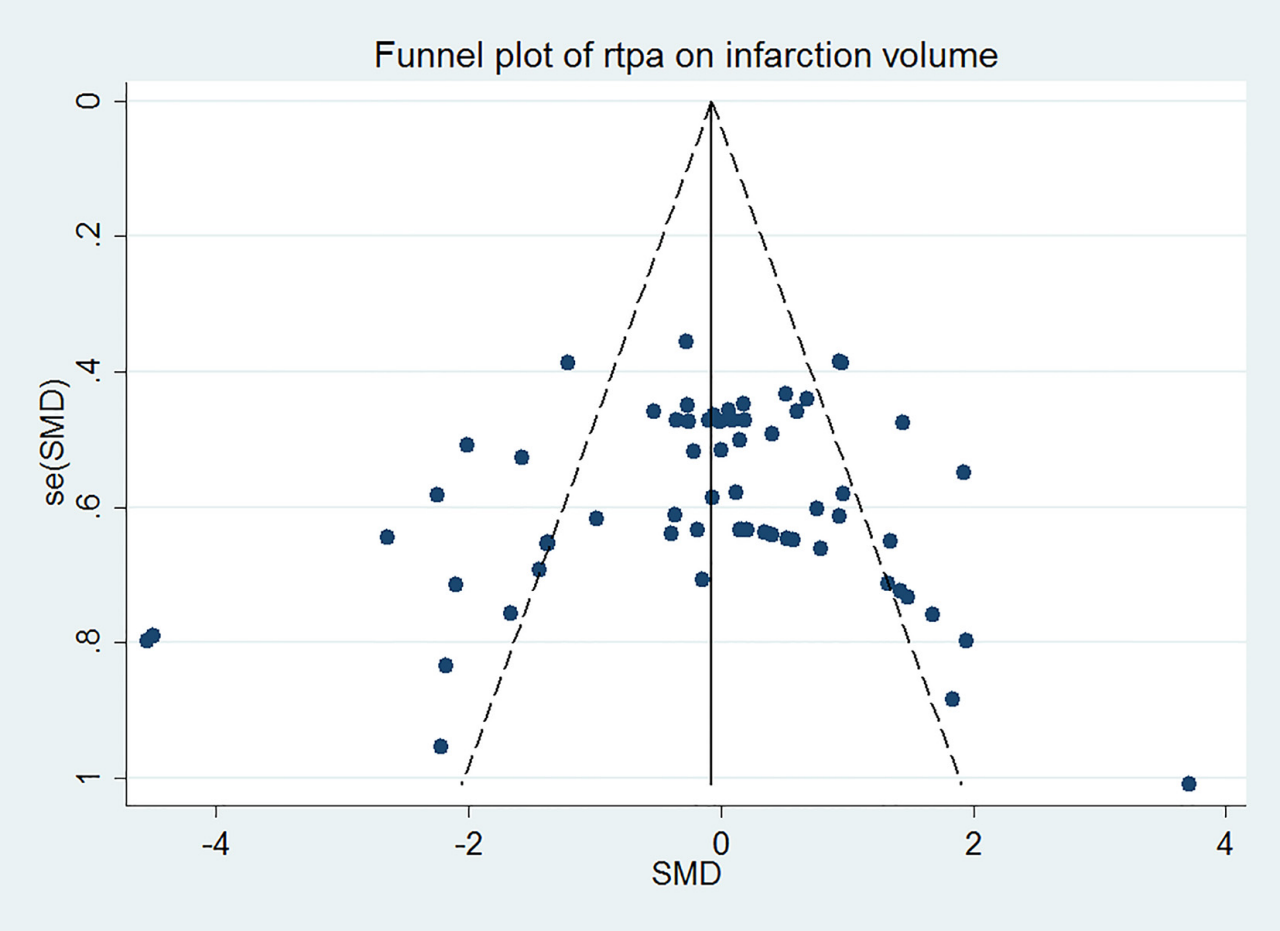
Funnel plot showing publication bias of rtPA’s effect on infarction volume. The funnel plot was nearly symmetrical by visual inspection and no significant publication bias was detected by Egger’s test (*p* = 0.276).

The effect of rtPA on infarction volume used in each study was provided in Fig 2 and no significantly positive effect was found as the total pooled SMD was -0.12 (95% confidence interval (CI), -0.39 to 0.15). However, there was high heterogeneity (*I*^*2*^ = 74%), afterwards, meta-regression (Table 3) and subgroup analyses (Table 4) were used to determine the potential sources of heterogeneity. STAIR score seemed to be the most important heterogeneity source from meta-regression analyses (p = 0.063) but can only account for 0.82% of heterogeneity exhibited in the subgroup analyses. None of species, model, duration of rtPA, timing of rtPA, dose of rtPA administration, timing of assessment and evaluation methodology was the source of heterogeneity. Sensitivity analyses demonstrated that the relationship between rtPA and infarction volume remained persistent after applying the leave-one-out method (Fig 3). The funnel plot was nearly symmetrical by visual inspection (Fig 4), and no significant publication bias was detected by Egger’s test (*p* = 0.276).

**Table 3:** Meta-regression results of rtPA’s effect on infarction volume.

	*Coef*.	*Std*.*Err*	*z*	*p*
***Species***	*-0*.*513*	*0*.*335*	*-1*.*53*	*0*.*131*
***Model***	*0*.*444*	*0*.*493*	*0*.*90*	*0*.*372*
***DI***	*0*.*001*	*0*.*002*	*0*.*39*	*0*.*698*
***Time***	*0*.*001*	*0*.*002*	*0*.*71*	*0*.*479*
***TA***	*-0*.*002*	*0*.*006*	*-0*.*25*	*0*.*804*
***Dose***	*0*.*012*	*0*.*047*	*0*.*25*	*0*.*802*
***STAIR score***	*0*.*276*	*0*.*146*	*1*.*89*	*0*.*063*
***Methodology***	*-0*.*103*	*0*.*169*	*-0*.*61*	*0*.*545*

DI, duration of ischemia; Time, timing of rtPA; TA, timing of assessment; Dose, dose of rtPA administration; STAIR, Stroke Therapy Academic Industry Roundtable; Methodology, evaluation methodology, Coef, coefficient; Std. Err, standard error; z, effect size.

**Table 4:** Subgroup meta-analysis results of rtPA’s effect on infarction volume.

*Subgroups*	*No*. *of studies (animals)*	*SMDs (95% CI)*	*Within-group heterogeneity*			*Effect of subgroup*		
			*Chi*^*2*^ *within*	*p-value*	*%variance explained*^a)^	*Chi*^*2*^ *between*	*p-value*	*%variance explained*^b)^
***Species***								
*rat*	*34 (495)*	*0*.*10 (-0*.*17 to 0*.*37)*	*65*.*59*	*0*.*00*	*50%*			
*mouse*	*31 (517)*	*-0*.*39 (-0*.*87 to 0*.*09)*	*170*.*77*	*0*.*00*	*82%*	*3*.*11*	*0*.*08*	*1*.*27%*
***Ischemic model***								
*filament*	*57 (921)*	*-0*.*13 (-0*.*43 to 0*.*18)*	*239*.*98*	*0*.*00*	*77%*			
*ligation*	*8 (91)*	*-0*.*03 (-0*.*45 to 0*.*39)*	*5*.*08*	*0*.*65*	*0%*	*0*.*13*	*0*.*72*	*0*.*05%*
***Duration of ischemia***								
*permenant*	*4 (68)*	*0*.*06 (-0*.*62 to 0*.*73)*	*5*.*15*	*0*.*16*	*42%*			
*transient*	*61 (944)*	*-0*.*13 (-0*.*41 to 0*.*16)*	*239*.*16*	*0*.*00*	*75%*	*0*.*25*	*0*.*62*	*0*.*10%*
***Timing of rtPA***								
*< = 180 min*	*48 (752)*	*-0*.*24 (-0*.*60 to 0*.*12)*	*225*.*34*	*0*.*00*	*79%*			
*180~270 min*	*3 (58)*	*0*.*63 (-0*.*18 to 1*.*44)*	*3*.*98*	*0*.*14*	*50%*			
*270~360 min*	*14 (210)*	*0*.*04 (-0*.*23 to 0*.*32)*	*7*.*05*	*0*.*90*	*0%*	*4*.*06*	*0*.*13*	*1*.*66%*
***Dose of rtPA***								
*<10mg/kg*	*26 (425)*	*-0*.*36 (-0*.*90 to 0*.*18)*	*146*.*13*	*0*.*00*	*83%*			
*10mg/kg*	*39 (587)*	*0*.*04 (-0*.*24 to 0*.*32)*	*92*.*76*	*0*.*00*	*59%*	*1*.*68*	*0*.*19*	*0*.*69%*
***Timing of assessment***								
*< = 24h*	*57 (891)*	*-0*.*16 (-0*.*45 to 0*.*13)*	*217*.*60*	*0*.*00*	*74%*			
*>24h*	*8 (121)*	*-0*.*12 (-0*.*64 to 0*.*89)*	*25*.*64*	*0*.*00*	*73%*	*0*.*46*	*0*.*50*	*0*.*19%*
***Evaluation methodology***								
*TTC*	*46 (720)*	*-0*.*18 (-0*.*53 to 0*.*17)*	*200*.*81*	*0*.*00*	*78%*			
*HE*	*3 (35)*	*0*.*52 (-0*.*18 to 1*.*21)*	*0*.*78*	*0*.*68*	*0%*			
*cresyl violet*	*11 (197)*	*0*.*31 (-0*.*12 to 0*.*74)*	*20*.*9*	*0*.*02*	*52%*			
*MAP-2 antibody*	*5 (60)*	*-1*.*19 (-1*.*76 to -0*.*62)*	*0*.*26*	*0*.*99*	*0%*	*20*.*69*	*0*.*00*	*8*.*44%*
***STAIR score***								
*< = 3*	*30 (450)*	*-0*.*35 (-0*.*85 to 0*.*15)*	*152*.*76*	*0*.*00*	*81%*			
*> = 4*	*35 (562)*	*0*.*06 (-0*.*22 to 0*.*34)*	*85*.*11*	*0*.*00*	*60%*	*2*	*0*.*16*	*0*.*82%*

A positive value of SMD means that rtPA has enlarged infarction volume and presents side effect after ischemic stroke.

SMD, standardized means difference; CI, confidence interval; min, minute; TTC, 2,3,5-triphenyltetrazolium chloride; HE, hematoxylin eosin staining; STAIR, Stroke Therapy Academic Industry Roundtable.

a) Percentage of variance within group explained by heterogeneity is given by *I*^*2*^.

b) Percentage of variance explained by moderator variable is given by Chi^2^ between/Chi^2^ total, where Chi^2^ total = 245.09.

### Effect of endogenous tPA on infarction volume

**Fig 5:**
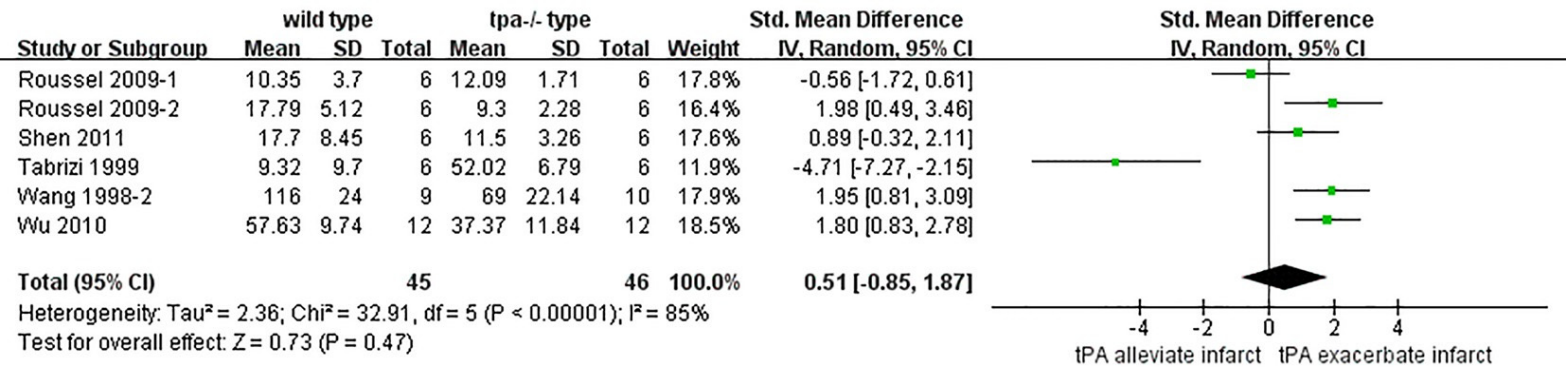
Forest plot of SMDs of endogenous tPA’s effect on infarction volume. The overall effect was not significant (*p* = 0.47) and heterogeneity was extremely high (*I*^*2*^ = 85%). SMD, standardized mean difference.

**Fig 6:**
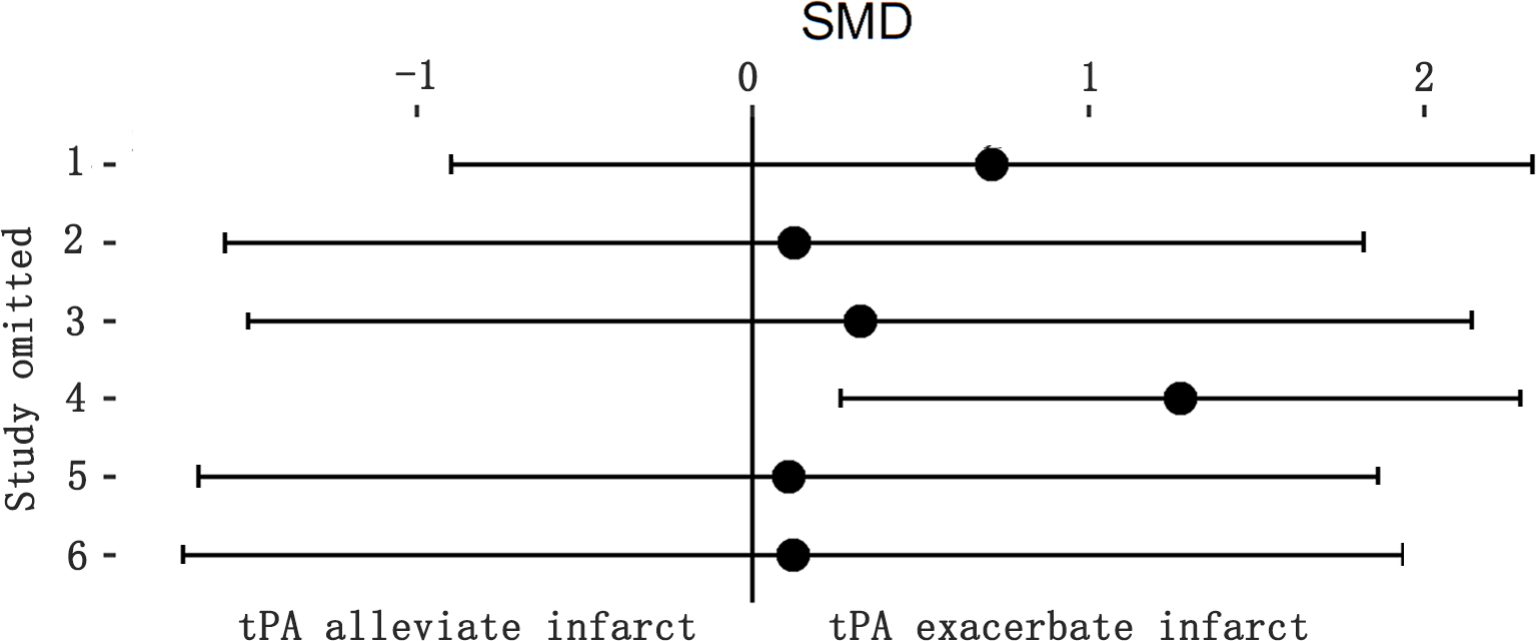
Sensitivity meta-analyses of endogenous tPA’s effect on infarction volume. The figure showed all 95%CI of SMDs after omitting each study as horizontal line. Endogenous tPA had enlarged infarction volume when leaving one study out. The result was unstable and need to be confirmed by expanding researches. CI, confidence interval; SMD, standardized mean difference.

**Fig 7:**
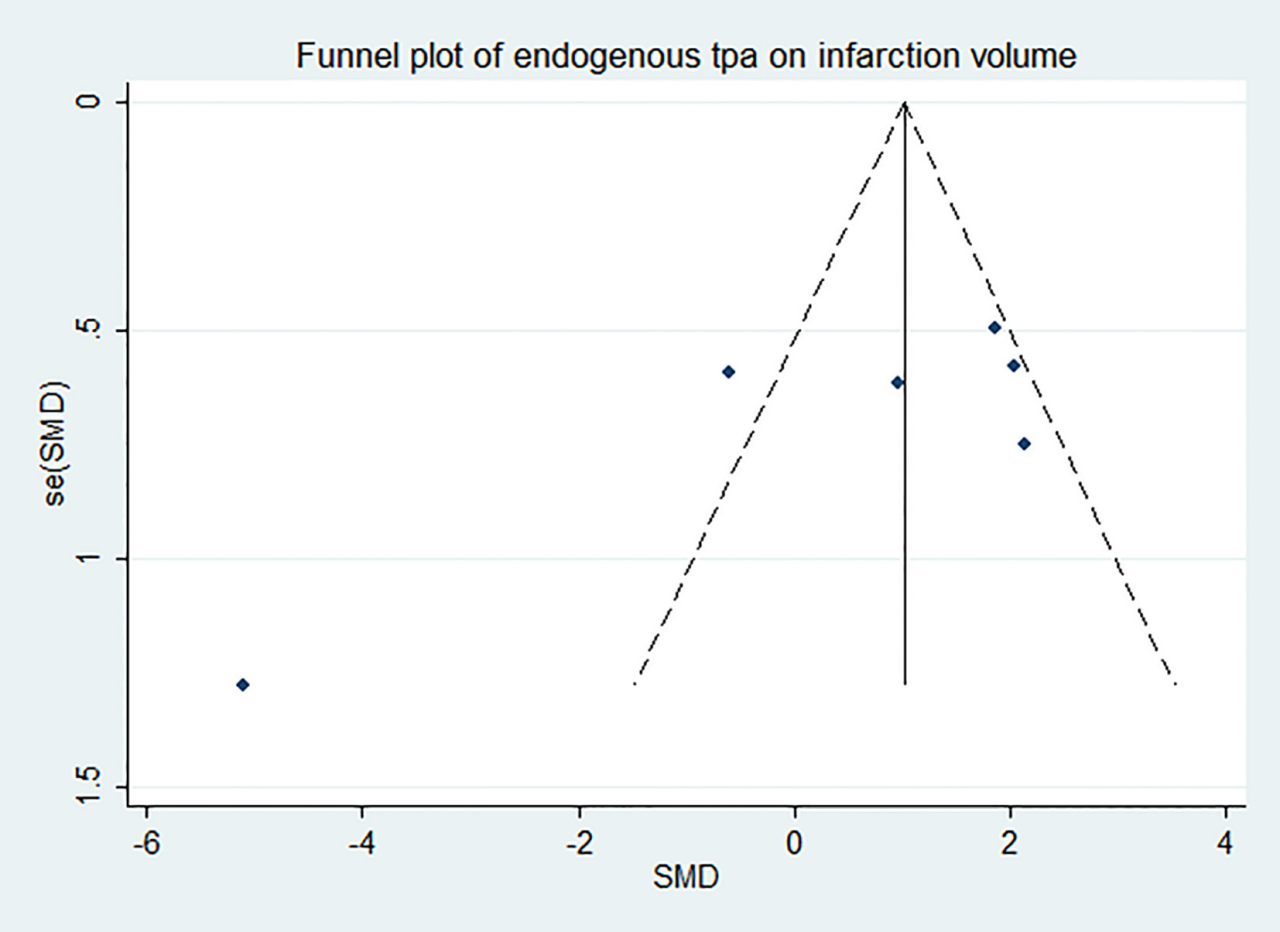
Funnel plot showing publication bias of endogenous tPA’s effect on infarction volume. The funnel plot was nearly symmetrical except one study and no significant publication bias was detected by Egger’s test (*p* = 0.120).

Five included studies in Fig 5 showed that there was no significantly positive effect of endogenous tPA on infarction volume (95%CI of SMD, -0.85 to 1.87) while the heterogeneity was extremely high (*I*^*2*^ = 85%). Subgroup meta-analyses were performed to determine the sources of heterogeneity but failed (Table 5). The effect size was unstable after excluding the study from Tabrizi[36] (Fig 6). More researches should be done to confirm the effect of endogenous tPA on infarction volume. The funnel plot was nearly symmetrical by visual inspection (Fig 7), and no significant publication bias was detected by Egger’s test (*p* = 0.120).

**Table 5:** Subgroup meta-analysis results of rtPA’s effect on infarction volume.

*Subgroups*	*No*. *of studies (animals)*	*SMDs (95% CI)*	*Within-group heterogeneity*			*Effect of subgroup*		
			*Chi*^*2*^ *within*	*p-value*	*%variance explained*^a)^	*Chi*^*2*^ *between*	*p-value*	*%variance explained*^b)^
***Ischemic model***								
*filament*	*4 (67)*	*0*.*37 (-1*.*48 to 2*.*21)*	*23*.*8*	*0*.*000*	*87%*			
*ligation*	*2 (24)*	*0*.*67 (-1*.*82 to 3*.*15)*	*6*.*92*	*0*.*009*	*86%*	*0*.*04*	*0*.*85*	*0*.*12%*
***Duration of ischemia***								
*permenant*	*3 (36)*	*0*.*72 (-0*.*70 to 2*.*13)*	*7*.*32*	*0*.*030*	*73%*			
*transient*	*3 (55)*	*0*.*01 (-2*.*70 to 2*.*72)*	*23*.*38*	*0*.*000*	*91%*	*0*.*2*	*0*.*65*	*1*.*98%*
***Evaluation methodology***								
*TTC*	*5 (79)*	*0*.*37 (-1*.*33 to 2*.*06)*	*32*.*89*	*0*.*000*	*88%*			
*HE*	*1 (12)*	*0*.*52 (-0*.*18 to 1*.*21)*	*-*	*-*	*-*	*0*.*25*	*0*.*62*	*1*.*88%*
***STAIR score***								
*< = 3*	*2 (43)*	*1*.*87 (1*.*12 to 2*.*61)*	*0*.*04*	*0*.*850*	*0%*			
*> = 4*	*4 (48)*	*-0*.*35 (-2*.*38 to 1*.*67)*	*22*.*47*	*0*.*000*	*87%*	*4*.*06*	*0*.*04*	*12*.*34%*

A positive value of SMD means that tPA deficient mice has decreased infarction volume and endogenous tPA presents neurotoxicity after ischemic stroke.

SMD, standardized means difference; CI, confidence interval; TTC, 2,3,5-triphenyltetrazolium chloride; HE, hematoxylin eosin staining; STAIR, Stroke Therapy Academic Industry Roundtable.

a) Percentage of variance within group explained by heterogeneity is given by *I*^*2*^.

b) Percentage of variance explained by moderator variable is given by Chi^2^ between/Chi^2^ total, where Chi^2^ total = 32.91.

### Effect of rtPA on BBB

**Fig 8:**
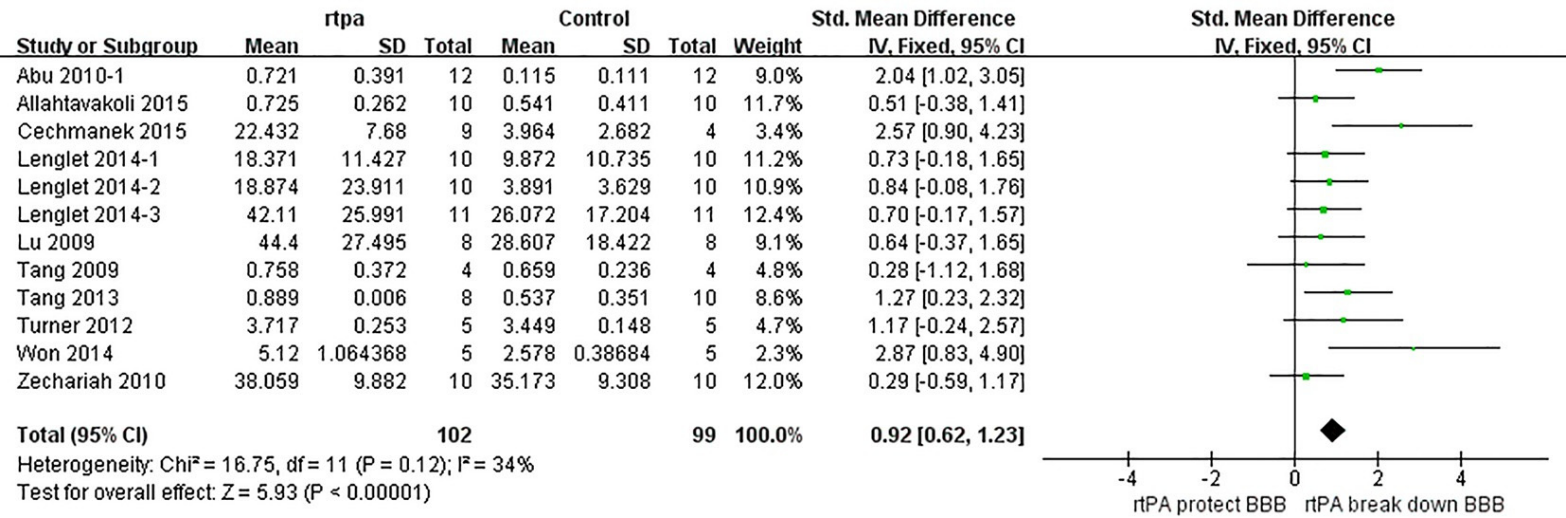
Forest plot of SMDs of rtPA’s effect on BBB. RtPA had significantly increased BBB permeability (95%CI of SMD, 0.62 to 1.23) and heterogeneity was extremely high (*I*^*2*^ = 85%). BBB, blood brain barrier; SMD, standardized mean difference; CI, confidence interval.

**Fig 9:**
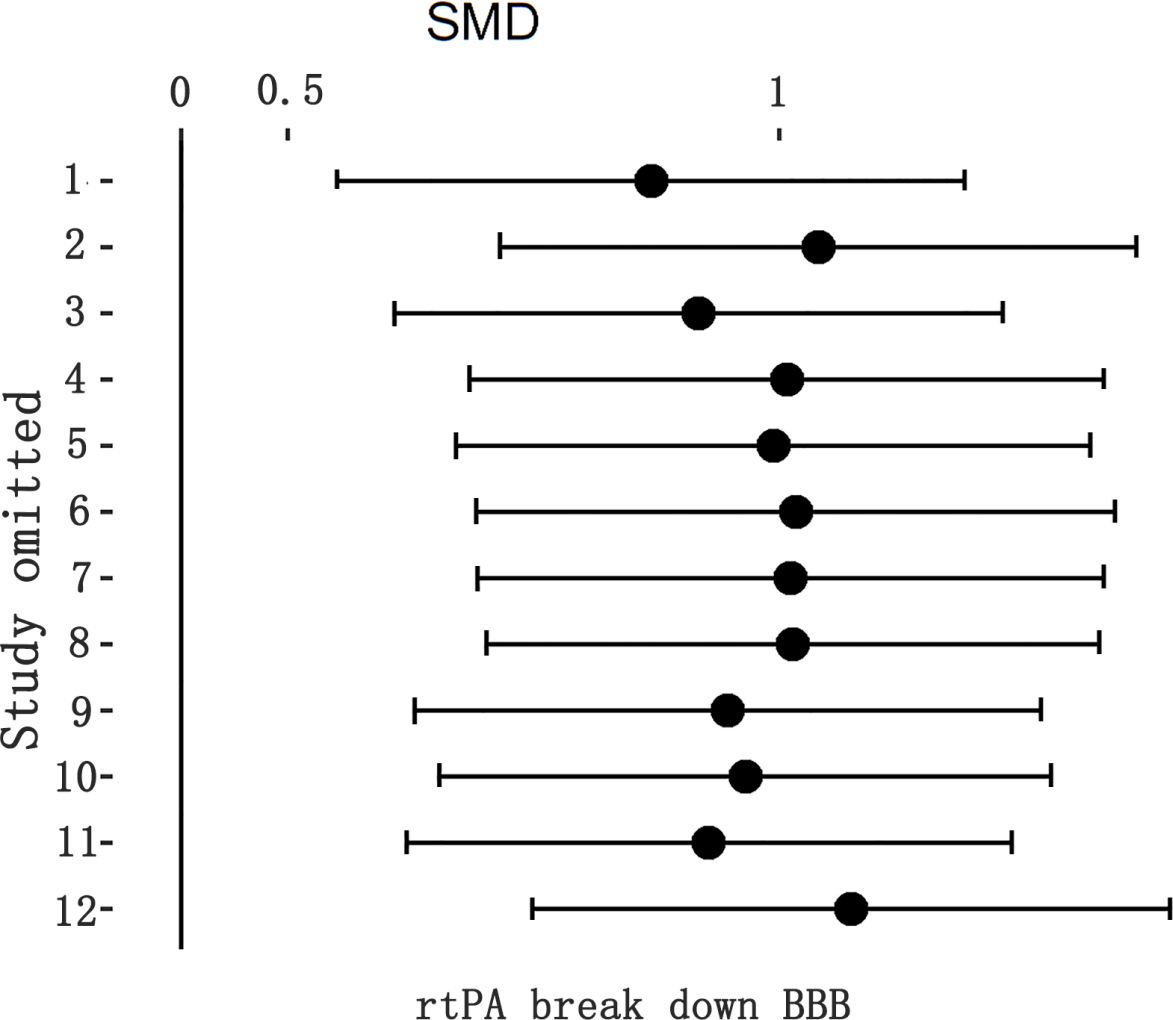
Sensitivity meta-analyses of rtPA’s effect on BBB. The figure showed all 95%CI of SMDs after omitting each study as horizontal line. The result stayed stable using leave-one-out method. BBB, blood brain barrier; CI, confidence interval; SMD, standardized mean difference.

**Fig 10:**
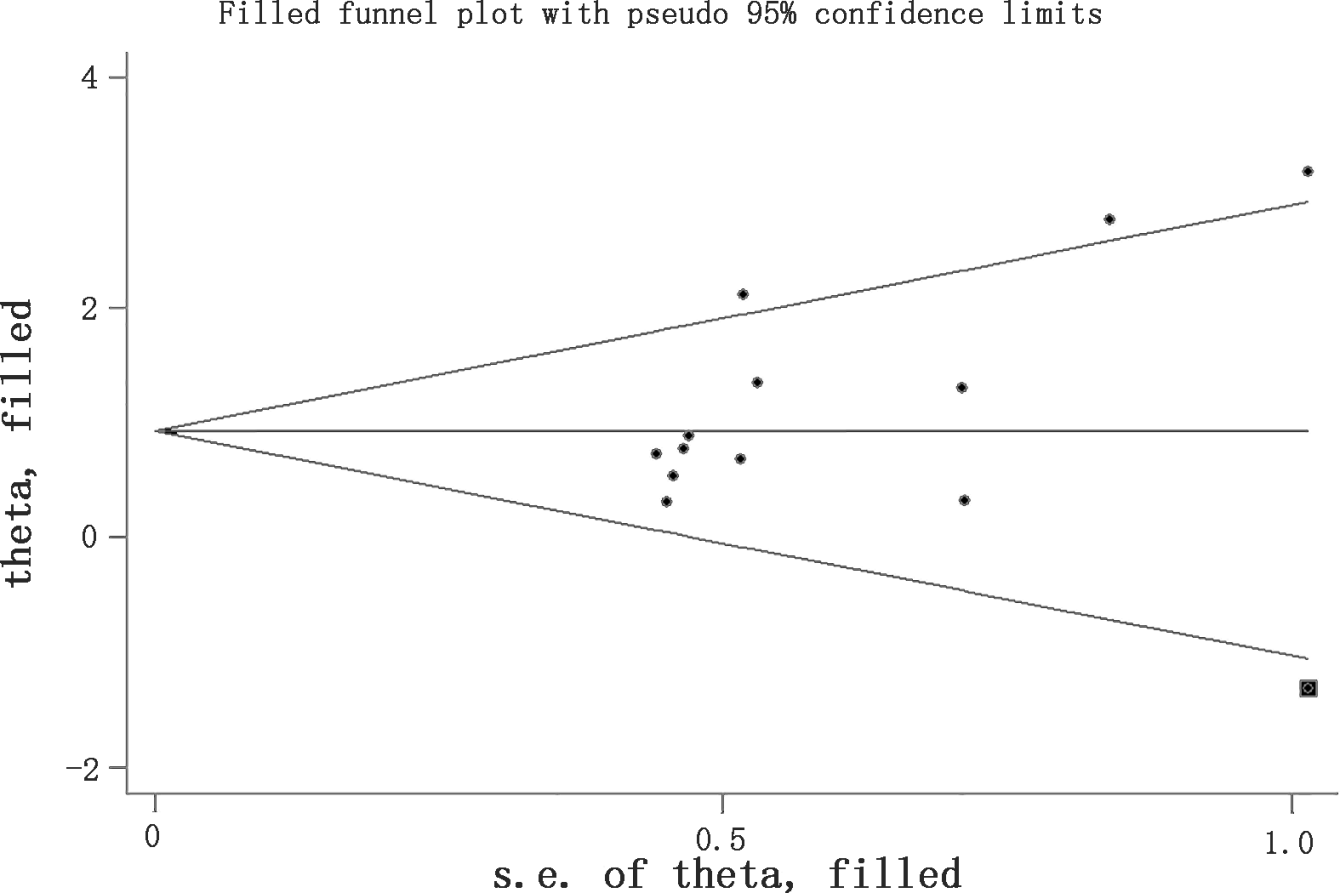
Filled funnel plot of rtPA’s effect on BBB using Trim and Fill method. Although there was significant publication bias detected by Egger’s test (*p* = 0.022), the result stayed stable after filling a study in square. BBB, blood brain barrier.

The effect of rtPA on BBB used in some studies was provided in Fig 8 and the total pooled SMD was 0.92 (95%CI, 0.62 to 1.23). That meant rtPA disrupted blood-brain barrier and increased BBB permeability. The heterogeneity was moderate (*I*^*2*^ = 34%), and the effect was stable when sensitivity analyses were performed (Fig 9). The *p* value was 0.022 detected by Egger’s test, and Trim and Fill method was used showing that the result was stable after filling one study (Fig 10).

### Effect of rtPA on brain edema

**Fig 11:**
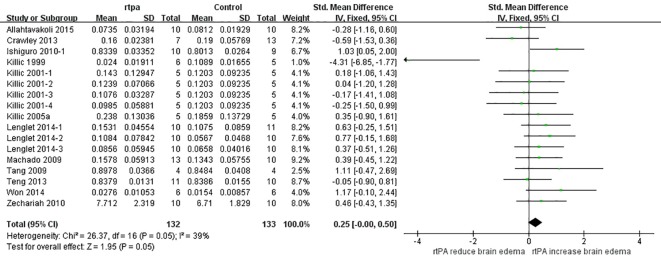
Forest plot of SMDs of rtPA’s effect on brain edema. RtPA had significantly exacerbated brain edema (95%CI of SMD, 0.00 to 0.50) and heterogeneity was moderate (*I*^*2*^ = 39%). SMD, standardized mean difference; CI, confidence interval.

**Fig 12:**
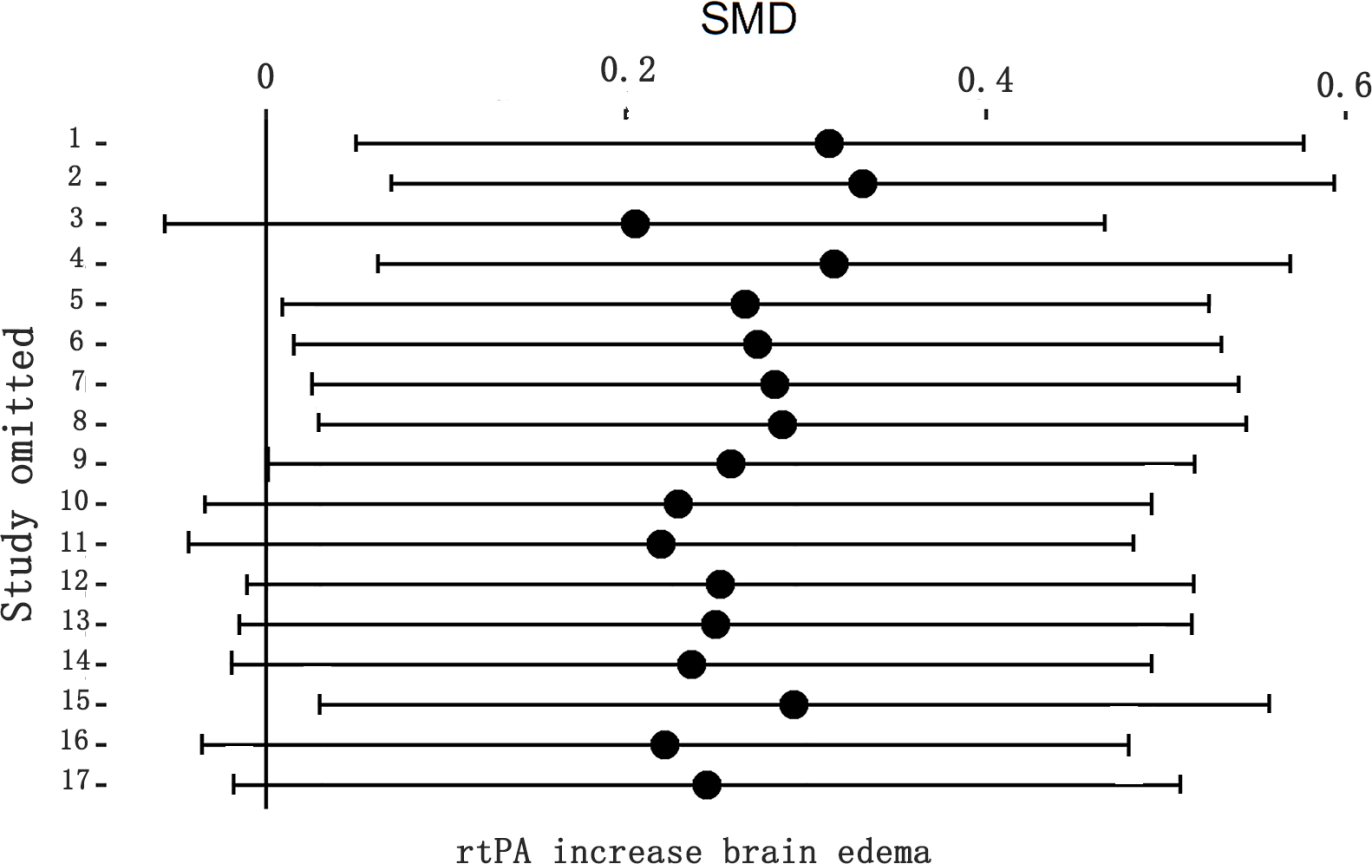
Sensitivity meta-analyses of rtPA’s effect on brain edema. The figure showed all 95% CI of SMDs after omitting each study as horizontal line. The result was unstable after leaving some studies out, which indicated that more researches need to be done to confirm the result. CI, confidence interval; SMD, standardized mean difference.

**Fig 13:**
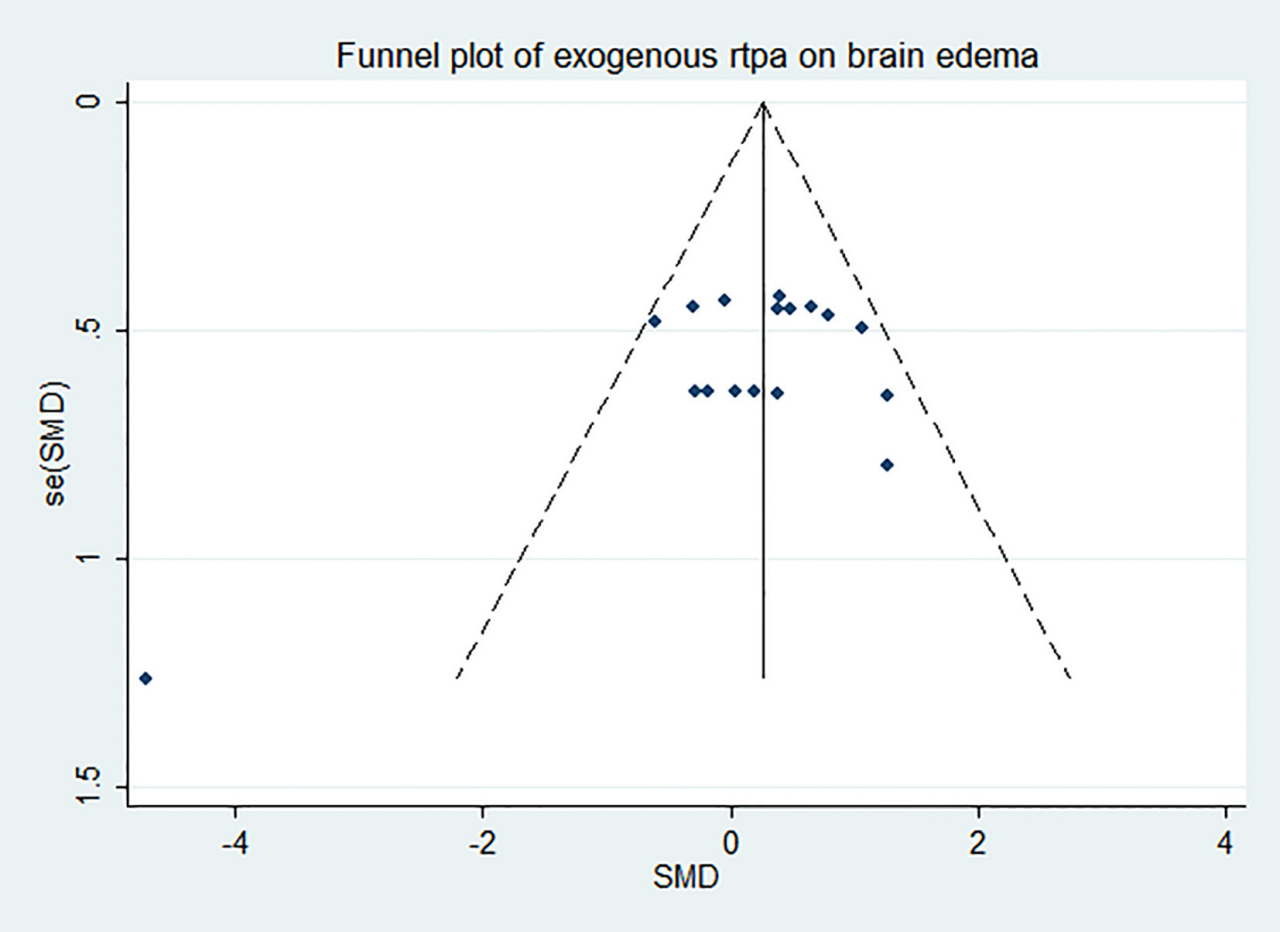
Funnel plot showing publication bias of rtPA’s effect on brain edema. The funnel plot was nearly symmetrical except one study by visual inspection and no significant publication bias was detected by Egger’s test (*p* = 0.140).

SMD analyses (Fig 11) showed that rtPA aggravated brain edema (95%CI, 0.00 to 0.50) and the heterogeneity was moderate (*I*^*2*^ = 39%). The result was unstable when sensitivity analyses were performed (Fig 12). More researches should be done to obtain more stable and reliable result. The funnel plot was nearly symmetrical by visual inspection (Fig 13), and no significant publication bias was detected by Egger’s test (*p* = 0.140).

### Effect of rtPA on intracerebral hemorrhage

**Fig 14:**
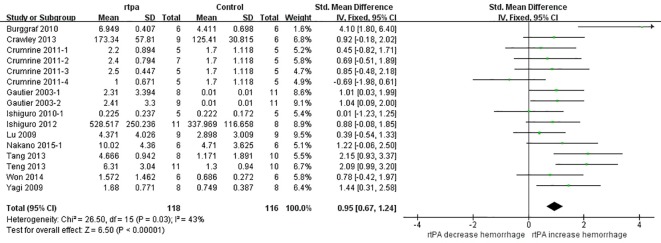
Forest plot of SMDs of rtPA’s effect on intracerebral hemorrhage. RtPA had significantly induced intracerebral hemorrhage (95%CI of SMD, 0.67 to 1.24) and heterogeneity was moderate (*I*^*2*^ = 43%). SMD, standardized mean difference; CI, confidence interval.

**Fig 15:**
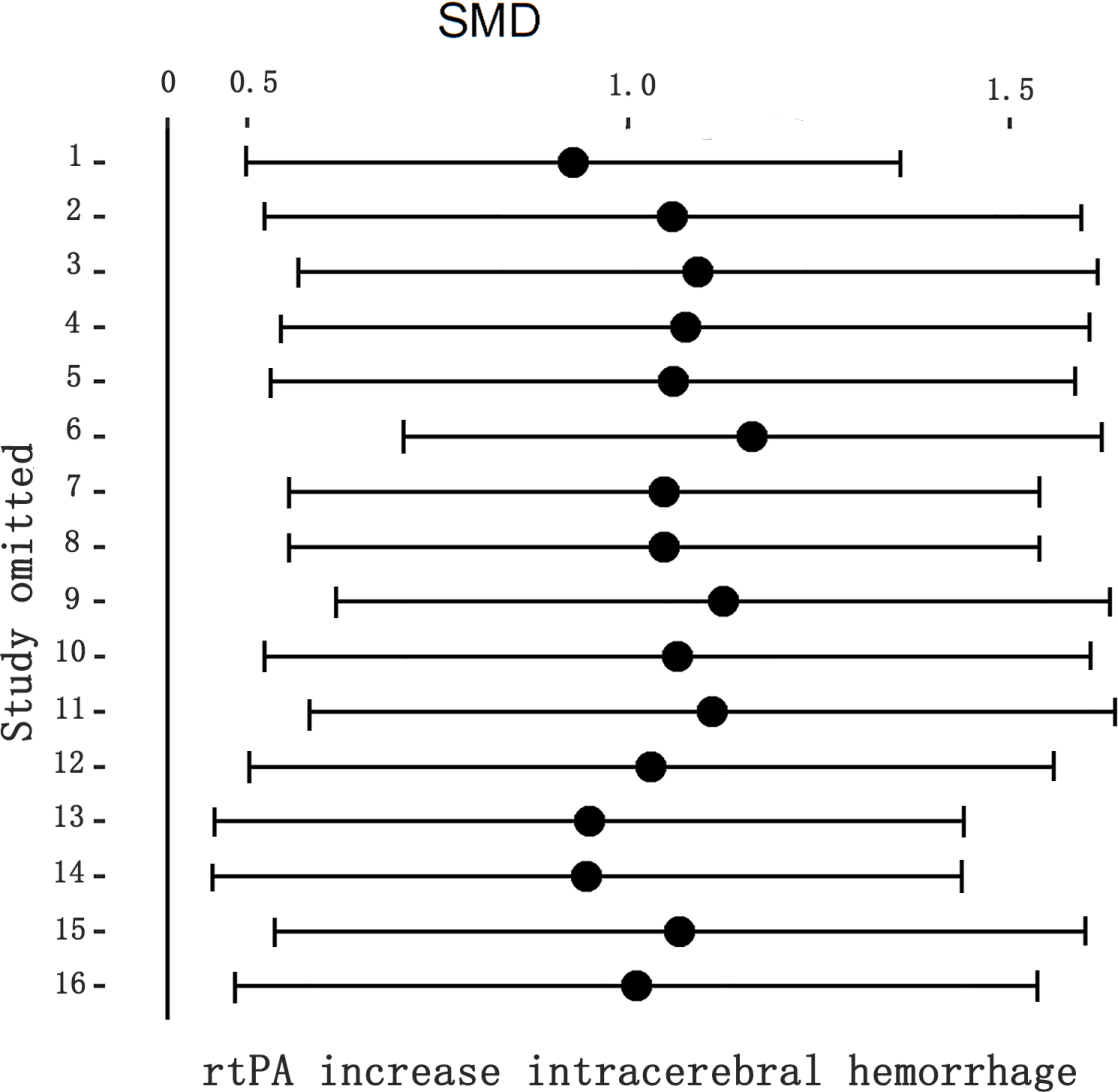
Sensitivity meta-analyses of rtPA’s effect on intracerebral hemorrhage. The figure showed all 95% CI of SMDs after omitting each study as horizontal line. The result was stable using the leave-one-out method. CI, confidence interval; SMD, standardized mean difference.

**Fig 16:**
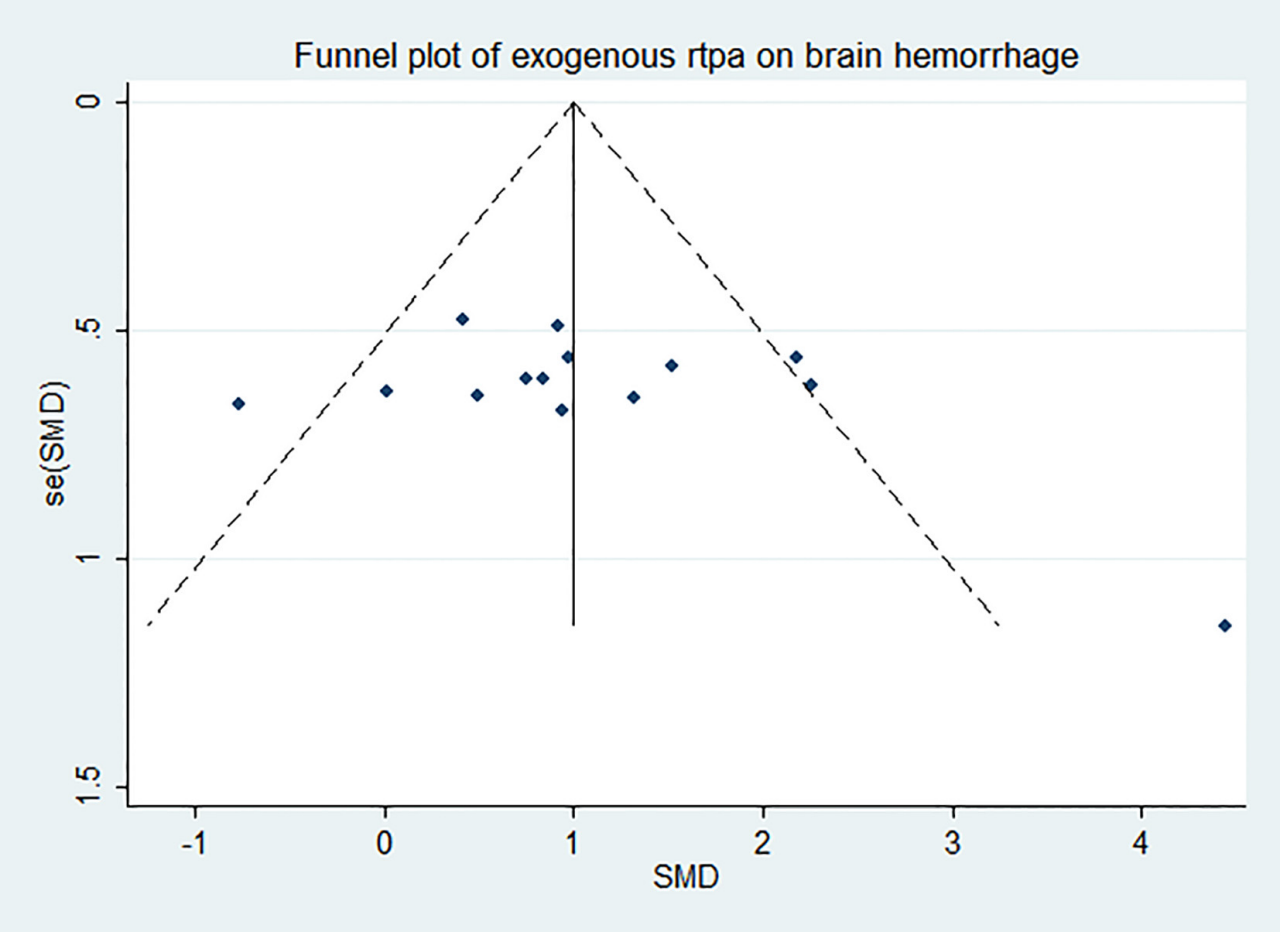
Funnel plot showing publication bias of rtPA’s effect on intracerebral hemorrhage. The funnel plot was nearly symmetrical by visual inspection and no significant publication bias was detected by Egger’s test (*p* = 0.179).

RtPA had significantly induced intracerebral hemorrhage, exhibited in Fig 14 (95%CI of SMD, 0.67 to 1.24). The heterogeneity (*I*^*2*^ = 43%) was moderate, and the effect was stable when sensitivity analyses were performed (Fig 15). The funnel plot was showed in Fig 16, and no significant publication bias was detected by Egger’s test (*p* = 0.179).

### Effect of rtPA on neurological function

**Fig 17:**
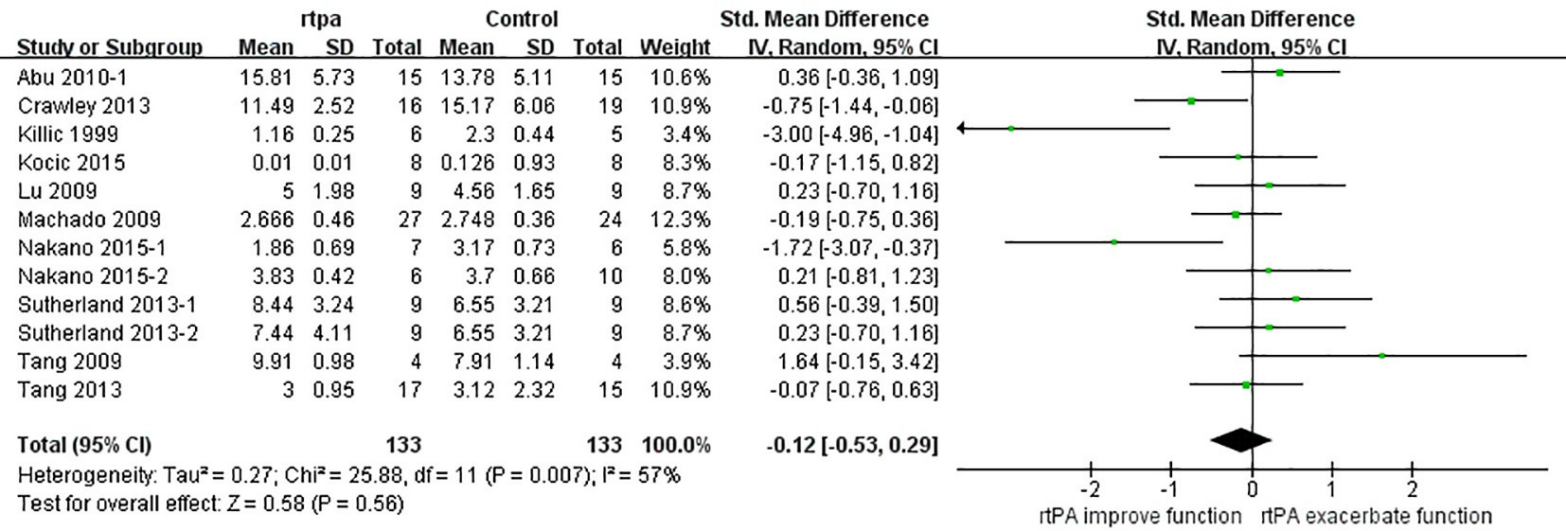
Forest plot of SMDs of rtPA’s effect on neurological function. The overall effect was not significant (*p* = 0.56) and heterogeneity was high (*I*^*2*^ = 57%). The result indicated that rtPA had no influence on neurological function of the survivals after mechanical stroke. SMD, standardized mean difference.

**Fig 18:**
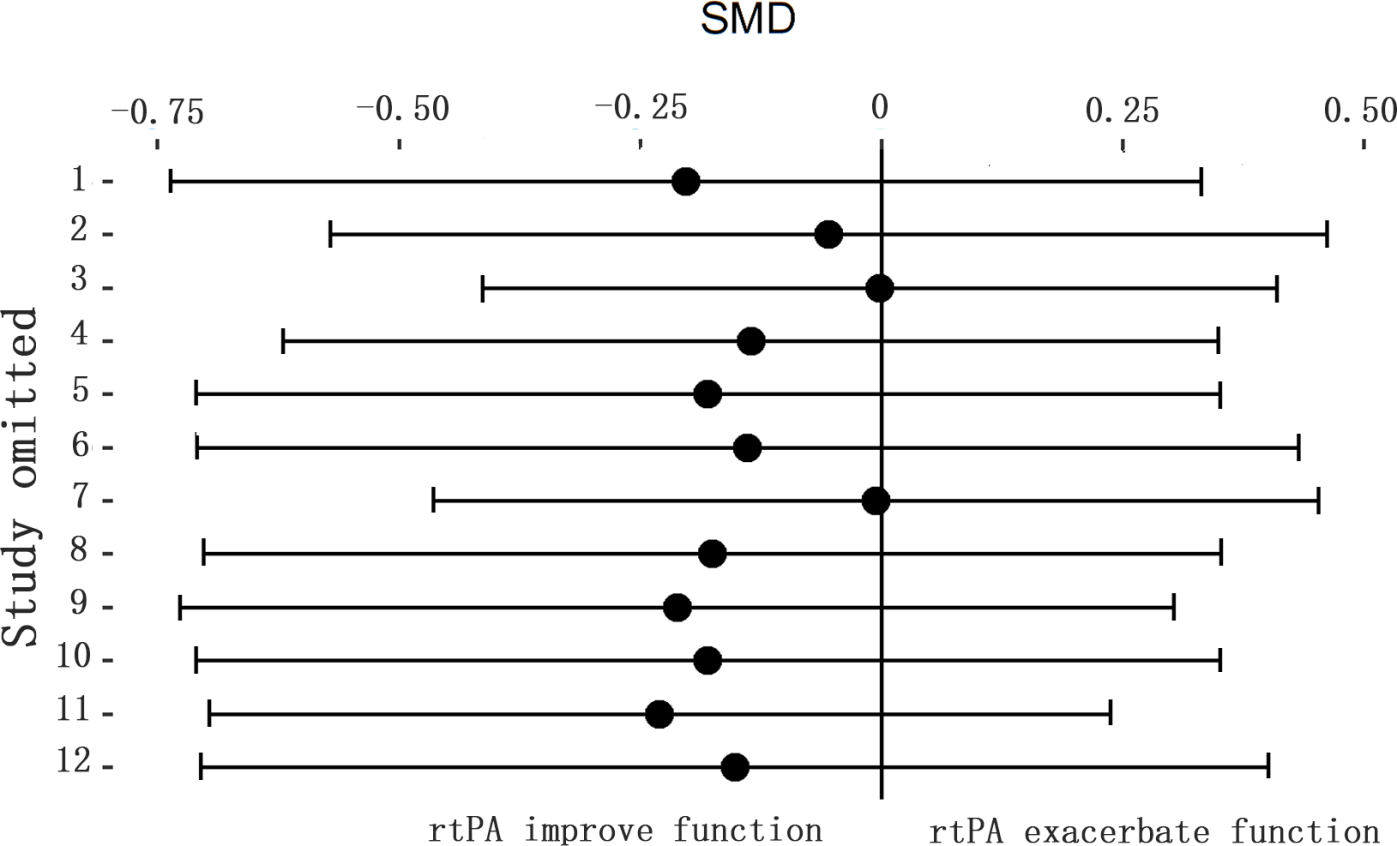
Sensitivity meta-analyses of rtPA’s effect on neurological function. The figure showed all 95% CI of SMDs after omitting each study as horizontal line. The result was stable using the leave-one-out method. CI, confidence interval; SMD, standardized mean difference.

**Fig 19:**
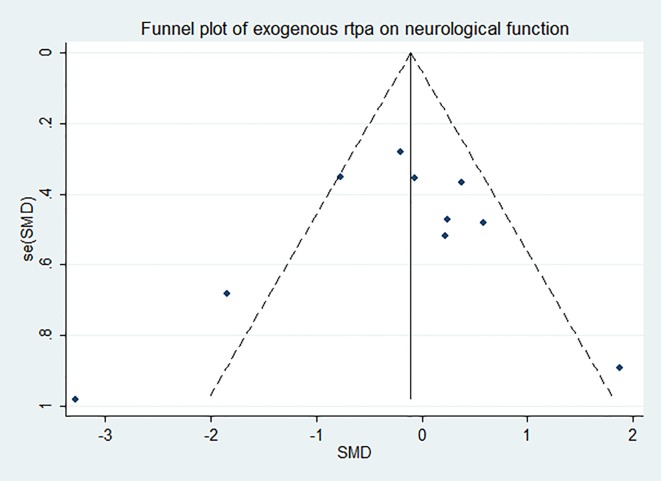
Funnel plot showing publication bias of rtPA’s effect on neurological function. The funnel plot was nearly symmetrical by visual inspection and no significant publication bias was detected by Egger’s test (*p* = 0.674).

The effect of rtPA on neurological deficit score used in some studies was provided in Fig 17 and rtPA had no significant effect on neurological function (95%CI of SMD, -0.53 to 0.29, *I*^*2*^ = 57%). Subgroup meta-analyses were obtained to detect potential moderator and the results were exhibited in Table 6. Species and STAIR score were the most important sources of heterogeneity, however, they can only account for no more than 20 percent. The effect was stable when sensitivity analyses were performed (Fig 18). The funnel plot was nearly symmetrical by visual inspection (Fig 19), and no significant publication bias was statistically detected by Egger’s test (*p* = 0.674).

**Table 6:** Subgroup meta-analysis results of rtPA’s effect on neurological function deficit.

*Subgroups*	*No*. *of studies (animals)*	*SMDs (95% CI)*	*Within-group heterogeneity*			*Effect of subgroup*		
			*Chi*^*2*^ *within*	*p-value*	*%variance explained*^a)^	*Chi*^*2*^ *between*	*p-value*	*%variance explained*^b)^
***Species***								
*rat*	*7 (159)*	*0*.*16 (-0*.*16 to 0*.*47)*	*5*.*66*	*0*.*46*	*0%*			
*mouse*	*5 (107)*	*-0*.*79 (-1*.*61 to 0*.*03)*	*13*.*2*	*0*.*01*	*70%*	*4*.*45*	*0*.*03*	*17*.*19%*
***Duration of ischemia***								
*permenant*	*1 (16)*	*-0*.*17 (-1*.*15 to 0*.*82)*	*-*	*-*	*-*			
*transient*	*11 (250)*	*-0*.*12 (-0*.*57 to 0*.*32)*	*25*.*86*	*0*.*00*	*61%*	*0*.*01*	*0*.*93*	*0*.*04%*
***Timing of rtPA***								
*< = 180 min*	*10 (232)*	*-0*.*20 (-0*.*68 to 0*.*28)*	*24*.*87*	*0*.*00*	*64%*			
*180~270 min*	*1 (16)*	*0*.*21 (-0*.*81 to 1*.*23)*	*-*	*-*	*-*			
*270~360 min*	*1 (18)*	*0*.*23 (-0*.*70 to 1*.*16)*	*-*	*-*	*-*	*0*.*97*	*0*.*62*	*3*.*75%*
***Dose of rtPA***								
*<10mg/kg*	*2 (48)*	*0*.*31 (-0*.*26 to 0*.*88)*	*0*.*05*	*0*.*82*	*0%*			
*10mg/kg*	*10 (218)*	*-0*.*23 (-0*.*71 to 0*.*25)*	*23*.*26*	*0*.*00*	*61%*	*2*.*02*	*0*.*16*	*7*.*81%*
***STAIR score***								
*< = 3*	*5 (107)*	*-0*.*69 (-1*.*52 to 0*.*14)*	*12*.*53*	*0*.*01*	*68%*			
*> = 4*	*7 (159)*	*0*.*13 (-0*.*30 to 0*.*57)*	*10*.*42*	*0*.*11*	*42%*	*2*.*94*	*0*.*09*	*11*.*36%*

A positive value of SMD means that rtPA has deteriorated neurological function after ischemic stroke.

SMD, standardized means difference; CI, confidence interval; STAIR, Stroke Therapy Academic Industry Roundtable.

a) Percentage of variance within group explained by heterogeneity is given by *I*^*2*^.

b) Percentage of variance explained by moderator variable is given by Chi^2^ between/Chi^2^ total, where Chi^2^ total = 25.88.

### Effect of rtPA on mortality rate

**Fig 20:**
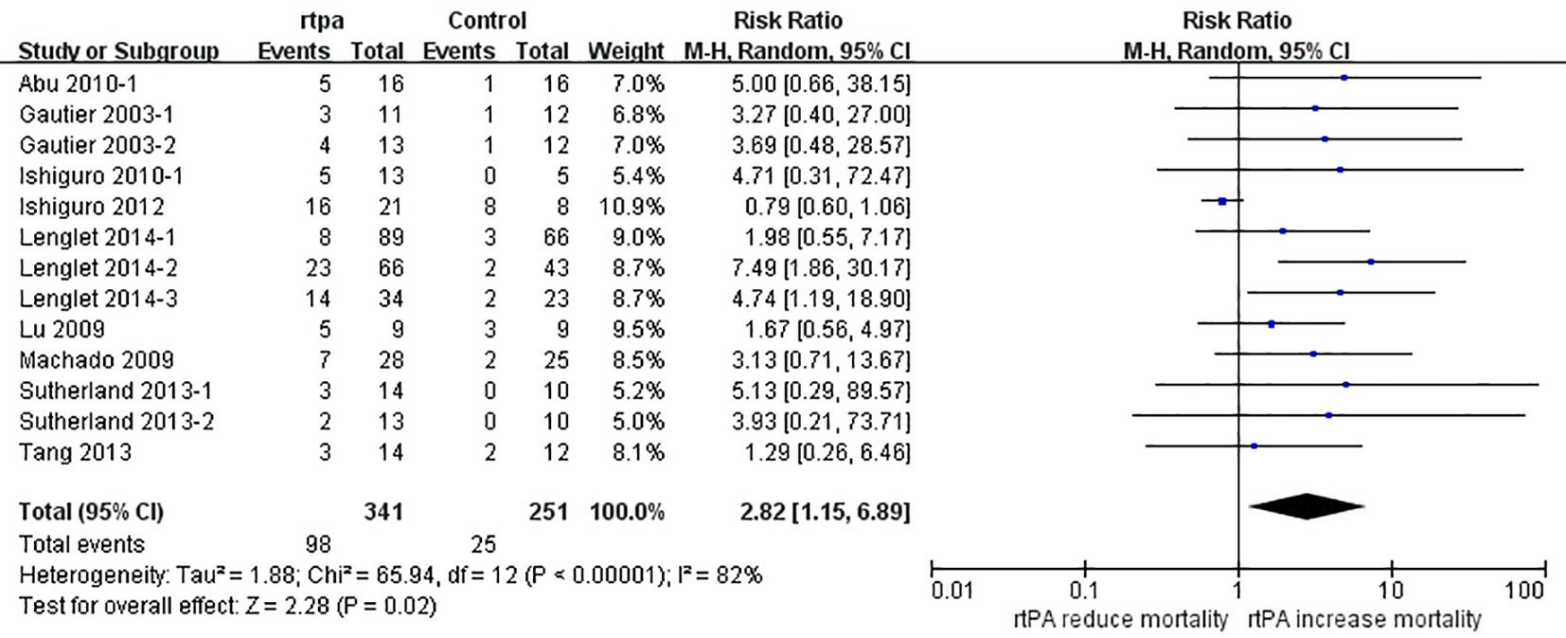
Forest plot of RRs of rtPA’s effect on mortality rate. RtPA had significantly increased mortality rate (95%CI of RR, 1.15 to 6.89) and heterogeneity was high (*I*^*2*^ = 82%). RR, risk ratio; CI, confidence interval.

**Fig 21:**
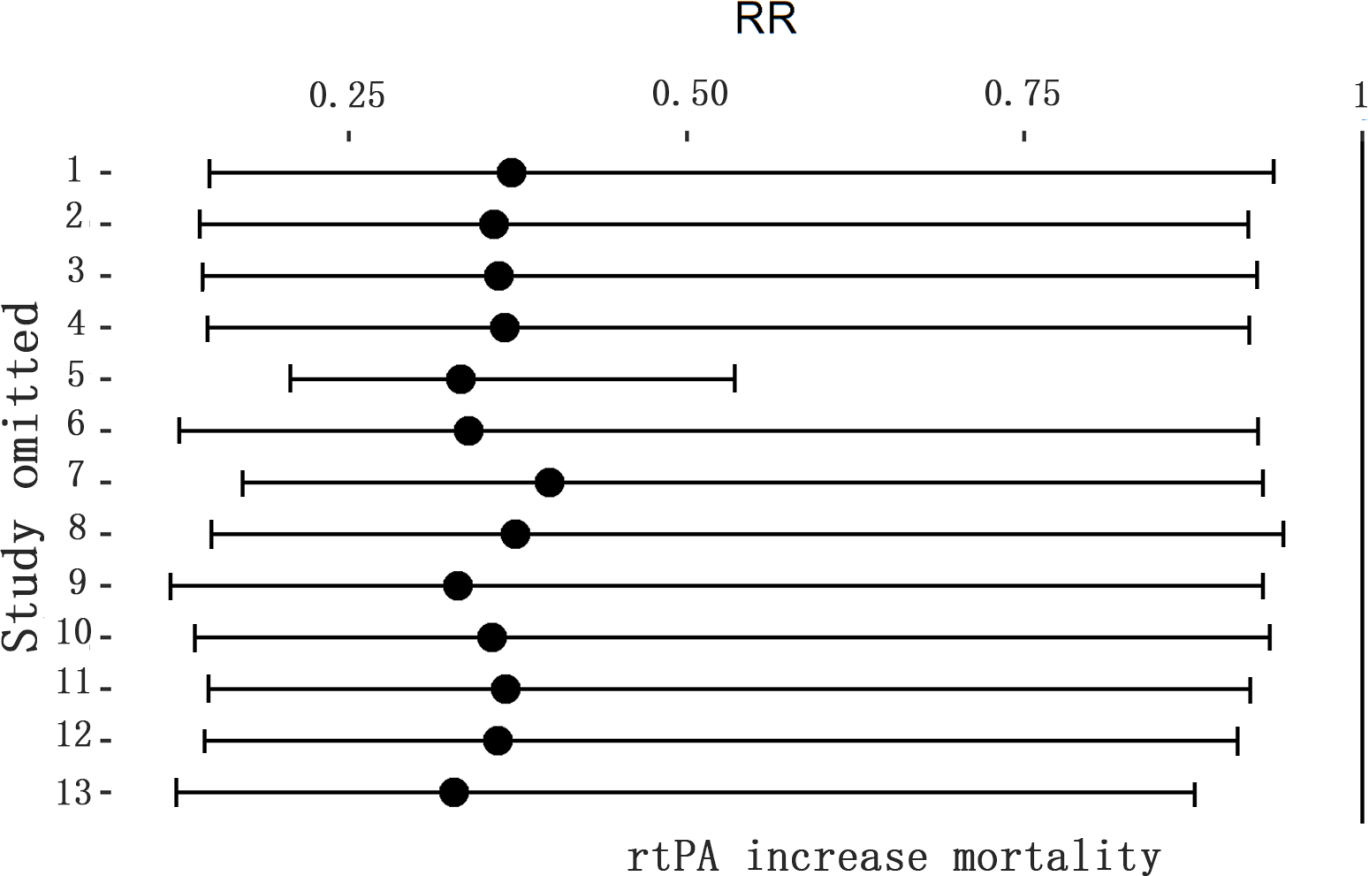
Sensitivity meta-analyses of rtPA’s effect on mortality rate. The figure showed all 95% CI of RRs after omitting each study as horizontal line. The result was stable using the leave-one-out method. CI, confidence interval; RR, risk ratio.

**Fig 22:**
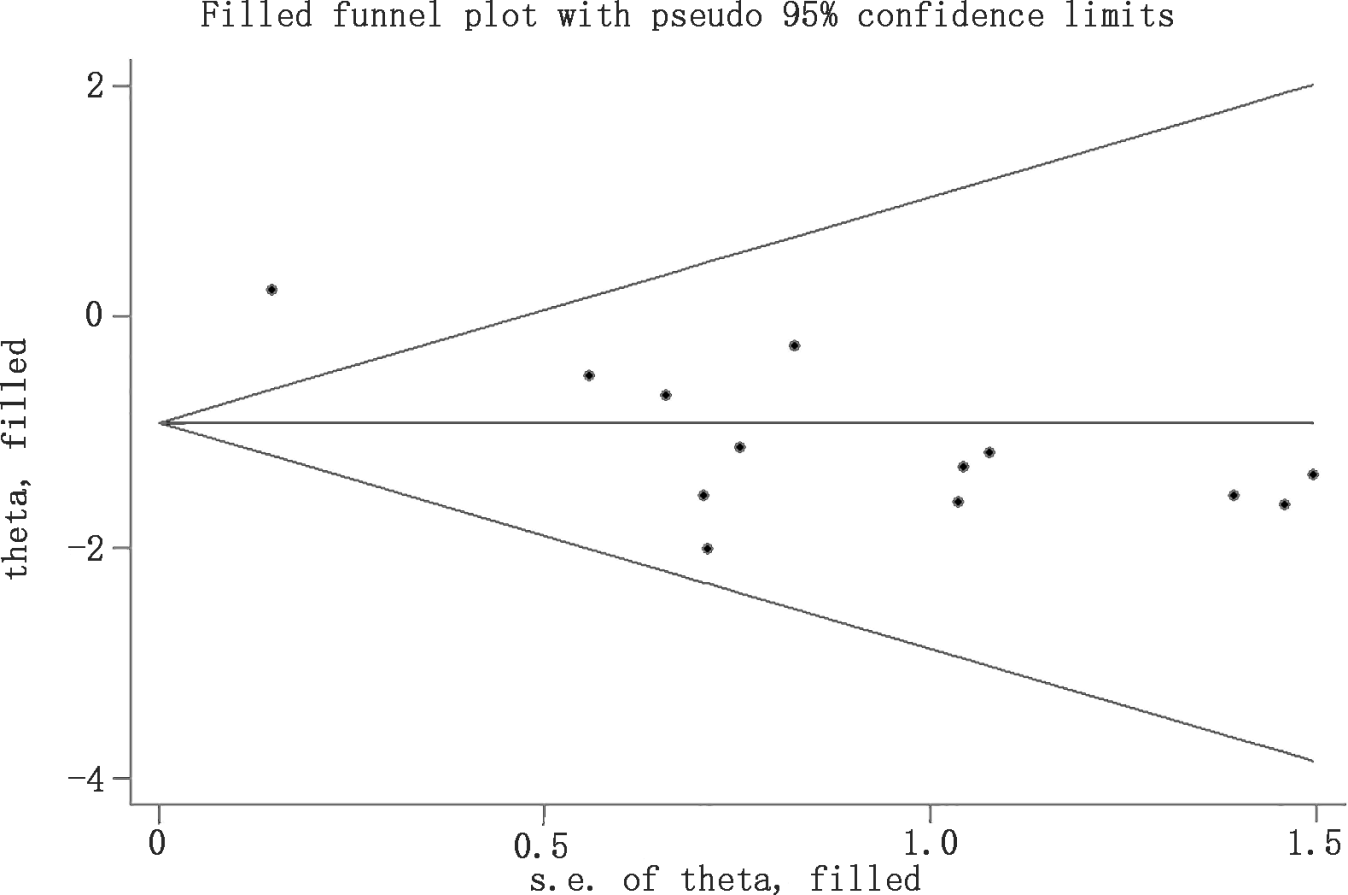
Filled funnel plot of rtPA’s effect on mortality rate using Trim and Fill method. Although there was significant publication bias detected by Egger’s test (*p* = 0.000), no extra study need to be filled and the result stayed stable after using Trim and Fill method.

The effect of rtPA on mortality used in some study was provided in Fig 20 and rtPA had significantly increased mortality rate in mechanical animal stroke (95%CI of RR, 1.15 to 6.89, *p* = 0.02). However, the heterogeneity was extremely high (*I*^*2*^ = 82%) but no source can be detected in subgroup analyses (Table 7). The result was stable in sensitivity meta-analyses (Fig 21). The *p* value was 0.000 detected by Egger’s test and the result hadn’t changed when analyzed by Trim and Fill method (Fig 22).

**Table 7:** Subgroup meta-analysis results of rtPA’s effect on mortality rate.

*Subgroups*	*No*. *of studies (animals)*	*RRs (95% CI)*	*Within-group heterogeneity*			*Effect of subgroup*		
			*Chi*^*2*^ *within*	*p-value*	*%variance explained*^a)^	*Chi*^*2*^ *between*	*p-value*	*%variance explained*^b)^
***Species***								
*rat*	*7 (198)*	*2*.*76 (1*.*42 to 5*.*39)*	*1*.*63*	*0*.*95*	*0%*			
*mouse*	*6 (394)*	*2*.*50 (0*.*52 to 12*.*13)*	*54*.*38*	*0*.*00*	*91%*	*0*.*01*	*0*.*91*	*0*.*02%*
***Duration of ischemia***								
*permenant*	*1 (26)*	*1*.*29 (0*.*26 to 6*.*46)*	*-*	*-*	*-*			
*transient*	*12 (566)*	*3*.*05 (1*.*14 to 8*.*17)*	*68*.*64*	*0*.*00*	*84%*	*0*.*8*	*0*.*37*	*0*.*56%*
***Timing of rtPA***								
*< = 180 min*	*8 (471)*	*3*.*97 (2*.*16 to 7*.*28)*	*2*.*24*	*0*.*95*	*0%*			
*180~270 min*	*0 (0)*	*-*	*-*	*-*	*-*			
*270~360 min*	*5 (121)*	*1*.*51 (0*.*58 to 3*.*89)*	*12*.*09*	*0*.*02*	*67%*	*2*.*84*	*0*.*09*	*4*.*31%*
***Dose of rtPA***								
*<10mg/kg*	*4 (99)*	*2*.*52 (1*.*09 to 5*.*79)*	*1*.*52*	*0*.*68*	*0%*			
*10mg/kg*	*9 (493)*	*2*.*71 (0*.*82 to 9*.*00)*	*60*.*4*	*0*.*00*	*87%*	*0*.*01*	*0*.*92*	*0*.*02%*
***STAIR score***								
*< = 3*	*2 (82)*	*1*.*45 (0*.*18 to 11*.*79)*	*7*.*86*	*0*.*01*	*87%*			
*> = 4*	*11 (510)*	*2*.*99 (1*.*81 to 4*.*96)*	*5*.*62*	*0*.*85*	*0%*	*0*.*43*	*0*.*51*	*0*.*65%*

A value of RR >1 means that rtPA has increased mortality rate after ischemic stroke.

RR, risk ratio; CI, confidence interval; min, minute; STAIR, Stroke Therapy Academic Industry Roundtable.

a) Percentage of variance within group explained by heterogeneity is given by *I*^*2*^.

b) Percentage of variance explained by moderator variable is given by Chi^2^ between/Chi^2^ total, where Chi^2^ total = 65.94

### Secondary outcomes of endogenous tPA

Limited studies had exhibited the effects of endogenous tPA on secondary efficacy outcomes and they can’t be merged together using meta-analysis. Only one study[50] presented the effect of endogenous tPA on BBB using Evans Blue dye extravasation and showed that endogenous tPA had significantly increased BBB permeability in 4 months old mice but not in 21 months old mice (n = 6, separately). Result from Tabrizi et al.[36] illustrated that brain edema was significantly increased by 2.3-fold in tPA deficient mice versus wild-type mice (n = 6, separately). However, Shen et al.[60] showed that neurological function measured by foot-fault and modified neurological severity score was significantly reduced in tPA deficient mice when compared to wild-type mice (n = 9, separately), while animal mortality rate between the two species was similar (about 40%).

## Discussion

Tissue plasminogen activator is a serine proteinase found not only in the intravascular space but also in a well-defined sub-set of neurons in the brain[19]. It is mainly secreted by endothelial cells and constitutes of five functional domains through which it interacts with different substrates, binds proteins and receptors[78, 79]. TPA can not only dissolve clot in the intravascular space but also display neuroprotective or neurotoxic effect in central nervous system[15, 20, 80]. It acts on considerable cellular pathways and mediates neuronal migration, neurite outgrowth and remodeling during development[78, 81] or in ischemic brain[82]. It is essential for long-term hippocampal plasticity[83]. However, it is reported that tPA can be rapidly released from neurons after exposure to hypoxia or hypoglycemia in vitro[19], then disrupts blood-brain barrier[84], activates microglia[85], and induces excitotoxic neuronal degeneration[12]. RtPA has already been widely used as a thrombolytic drug in acute ischemic stroke since 1996[86], and it is still controversial whether it is neuroprotective or neurotoxic besides its thrombolysis property.

This meta-analysis followed a former one performed by Harston GW[26] and developed it in some way. We had searched databases since 1980’s and a total of 47 studies were included finally. The two opposite viewpoints about the effect of tPA on cerebral infarction have argued with each other for several years. Some researchers owed it to different sources (exogenous and endogenous), or morphological structures (single chain (sc-tPA) and double chain (dc-tPA))[78]. They demonstrated that endogenous tPA displayed neuroprotective activities while exogenous rtPA was neurotoxic[20, 79]. Using primary cultures of mouse cortical neurons, Bertrand T demonstrated that sc-tPA was the only one capable to promote NMDAR-induced calcium influx and subsequent excitotoxicity, both sc-tPA and tc-tPA can activate epidermal growth factor receptors (EGFRs) to mediate neuroprotective effects of tPA[87]. Therefore, we analyzed effects of both rtPA and endogenous tPA on cerebral infarction.

Different from most researches, we found that exogenous rtPA had no effect on infarction volume while the heterogeneity had reached up to 74%. The result was stable in sensitivity analyses. Meta-regression and subgroup meta-analyses were used to determine the sources of heterogeneity but failed. Species can only account for 1.27% of heterogeneity, nor did model, duration of ischemia, timing of rtPA, dose of rtPA administration and STAIR score. Four evaluation methodologies were used to calculate infarction volume, and it can only explain 8.44% of the total heterogeneity. SMD analyses showed that endogenous tPA hadn’t influenced infarction volume either. However, the result was unstable perhaps due to limited obtainable researches and a final conclusion can’t be made arbitrarily. Meanwhile, studies about the secondary efficacy outcomes of endogenous tPA were too limited to systematic review, either.

BBB consists of vascular endothelial cells, basement membrane and endfeet. It is always reported to be disrupted after cerebrovascular disease, especially during reperfusion after thrombolysis. We found that rtPA had significantly increased BBB permeability, whilst the result was reliable and stable. It was reported that rtPA can upregulate brain metalloproteinases (MMPs) levels after focal cerebral ischemia[88, 89]. MMPs play important roles in rtPA-mediated injury, including tPA-LRP (Low-density-lipoprotein Receptor-related Protein), tPA-APC (Activated Protein C) /PAR1 (Protease Activated Receptor-1) and tPA-NMDAR (N-methyl-D-aspartate receptor) pathway[15, 84, 90]. MMPs are known to play crucial role in disrupting BBB due to their ability to digest endothelial basal lamina[91]. They are also involved in the pathogenesis of oxidative stress and inflammation. Niego. B. suggested that tPA can cause marked morphologic and functional changes in both brain endothelial cells and astrocytes via plasmin using an in vitro BBB model[92]. The risk of BBB disruption may contribute to more serious consequences such as brain edema and intracerebral hemorrhage[93]. We found that rtPA had significantly exacerbated brain edema although the result was unstable. RtPA can not only lead to angioedema through BBB disruption, but also result in cytotoxic brain edema through excitotoxic neurotoxicity[94].

RtPA had increased risk of intracerebral hemorrhage as well, probably due to BBB disruption. Intracerebral hemorrhage is the least treatable form of stroke and is associated with high morbidity and mortality from our former researches[95–97]. We wondered whether rtPA deteriorated neurological function or not, then neurological deficit score in acute phase was gathered and compared. The result showed that rtPA hadn’t influenced neurological function in animals after mechanical stroke at all. However, mortality rate of animals treated with rtPA had increased when compared with saline group. That was inconsistent with a former meta-analysis of randomized controlled clinical trials[98]. It was probably because that beneficial thrombolysis property was not considered in our study. RtPA increased mortality rate probably through disrupting BBB, aggravating brain edema and inducing intracerebral hemorrhage. Whether rtPA influences long-term neurological behavior besides its thrombolysis property, just as chronic cerebral ischemia[99], is worthy further researching.

There are several notable limitations to this study. Firstly, it is a preclinical meta-analysis but not a clinical meta-analysis of randomized controlled trial. Although a large number of animal experiments have been performed on this issue, the quantity of human study is so small that it is difficult to get rid of rtPA’s thrombolysis property in human study. Secondly, heterogeneity still existed, even though we tried to determine the source of heterogeneity. It was probably because that tPA’s effect was not a primary end point in some studies.

## Conclusions

This meta-analysis reveals that both endogenous tPA and rtPA haven’t enlarge infarction volume, or deteriorated survival’s neurological function. RtPA would disrupt blood-brain barrier, aggravate brain edema, induce intracerebral hemorrhage and increase mortality rate. We conclude that rtPA can lead to neurological side effect independent on thrombolysis in mechanical animal stroke, which may account for clinical exacerbation for stroke patients that do not achieve vascular recanalization with rtPA. A PRISMA checklist for this article follows in supporting information part as S1 Table.

## Supporting Information

S1 Table This is the PRISMA checklist for this article. (DOC)
